# Bacterial group II introns generate genetic diversity by circularization and *trans*-splicing from a population of intron-invaded mRNAs

**DOI:** 10.1371/journal.pgen.1007792

**Published:** 2018-11-21

**Authors:** Félix LaRoche-Johnston, Caroline Monat, Samy Coulombe, Benoit Cousineau

**Affiliations:** Department of Microbiology and Immunology, Microbiome and Disease Tolerance Centre (MDTC), McGill University, Montréal, Québec, Canada; Department of Chemistry and Biochemistry, University of California, UNITED STATES

## Abstract

Group II introns are ancient retroelements that significantly shaped the origin and evolution of contemporary eukaryotic genomes. These self-splicing ribozymes share a common ancestor with the telomerase enzyme, the spliceosome machinery as well as the highly abundant spliceosomal introns and non-LTR retroelements. More than half of the human genome thus consists of various elements that evolved from ancient group II introns, which altogether significantly contribute to key functions and genetic diversity in eukaryotes. Similarly, group II intron-related elements in bacteria such as abortive phage infection (Abi) retroelements, diversity generating retroelements (DGRs) and some CRISPR-Cas systems have evolved to confer important functions to their hosts. In sharp contrast, since bacterial group II introns are scarce, irregularly distributed and frequently spread by lateral transfer, they have mainly been considered as selfish retromobile elements with no beneficial function to their host. Here we unveil a new group II intron function that generates genetic diversity at the RNA level in bacterial cells. We demonstrate that Ll.LtrB, the model group II intron from *Lactococcus lactis*, recognizes specific sequence motifs within cellular mRNAs by base pairing, and invades them by reverse splicing. Subsequent splicing of ectopically inserted Ll.LtrB, through circularization, induces a novel *trans*-splicing pathway that generates exon 1-mRNA and mRNA-mRNA intergenic chimeras. Our data also show that recognition of upstream alternative circularization sites on intron-interrupted mRNAs release Ll.LtrB circles harboring mRNA fragments of various lengths at their splice junction. Intergenic *trans*-splicing and alternative circularization both produce novel group II intron splicing products with potential new functions. Overall, this work describes new splicing pathways in bacteria that generate, similarly to the spliceosome in eukaryotes, genetic diversity at the RNA level while providing additional functional and evolutionary links between group II introns, spliceosomal introns and the spliceosome.

## Author summary

A hallmark of eukaryotic cells is the ability of their numerous introns, sequences that interrupt genes, to generate genetic diversity through the expression of several different protein variants from a single gene. These eukaryotic introns share a common ancestor with bacterial group II introns, which are mobile, scarce and maintained at low copy-levels. Because of this stark contrast, bacterial group II introns are considered as selfish mobile elements with no beneficial function to their hosts. As a challenge to this longstanding view, we describe here a new group II intron function that expands the genetic diversity and overall complexity of its bacterial host transcriptome. This ability of combining disparate mRNA fragments expands the repertoire of functional products at the disposal of the bacterial host. Our work thus provides a potential explanation of why group II introns were maintained in bacteria over the course of evolution, despite strong selective pressure to streamline their genomes. Moreover, generating new combinations of RNA molecules as a by-product of their otherwise selfish mobility pathway provides a new evolutionary link into how group II introns later evolved to fully support this function in the nuclei of eukaryotes.

## Introduction

Bacterial group II introns are large RNA enzymes that mostly behave as retromobile elements [1–5]. Following their autocatalytic excision from interrupted RNA transcripts, they can reinsert within identical or similar DNA target sequences by retrohoming or retrotransposition, respectively [6–8]. These retromobile genetic elements are present in archaea, bacteria, and bacterial-derived organelles such as plant and fungal mitochondria, and plant chloroplasts [9]. While group II introns are somewhat infrequent in archaea, roughly one quarter of all sequenced bacterial genomes harbor one to a few copies displaying a broad phylogenetic distribution in the bacterial kingdom [10]. In sharp contrast, no functional group II introns were yet described in the nuclear genome of eukaryotes where they seem to be functionally excluded [11]. Although mitochondrial and chloroplastic group II introns mainly interrupt housekeeping genes, bacterial group II introns are generally found in non-coding sequences and associated with other mobile genetic elements [5]. Organellar group II introns thus primarily function as classic intervening sequences while bacterial group II introns behave like mobile elements. Bacterial group II introns were also shown to propagate by conjugation within and between species, invading the chromosome or resident plasmids of their new hosts using either the retrohoming or retrotransposition pathways [12–14].

**Fig 1:**
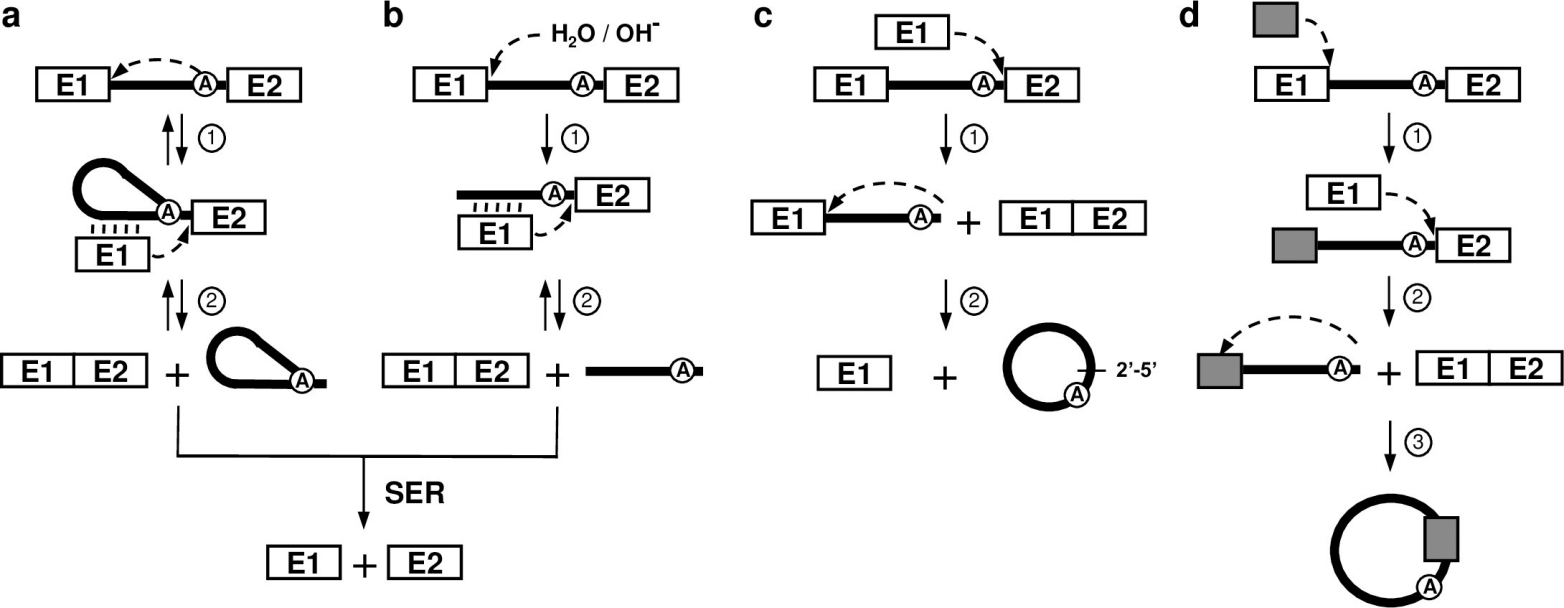
Group II intron splicing pathways. (**a**) Branching pathway. Following transcription of the interrupted gene, the 2´-OH residue of the branch-point nucleotide (A) initiates the first nucleophilic attack at the exon 1-intron junction (step 1). This transesterification reaction connects the 5´ end of the intron to the branch point and releases exon 1 that remains associated to the intron through base pairing interactions (EBS-IBS interactions) (vertical lines). The liberated 3´-OH at the end of exon 1 then initiates a second nucleophilic attack at the intron-exon 2 junction (step 2), ligating the two exons and releasing the intron as a lariat. (**b)** Hydrolytic pathway. A hydroxyl ion or a water molecule initiates the first nucleophilic attack at the exon 1-intron junction (step 1). The second nucleophilic attack at the intron-exon 2 junction is initiated by the liberated 3´-OH at the end of exon 1 (step 2) which ligates the two exons and releases a linear intron. (**c**) Circularization pathway. The first nucleophilic attack takes place at the intron-exon 2 junction and is initiated by the 3´-OH of a free exon 1 (step 1) generating ligated exons and a circularization intermediate where the linear intron is still attached to exon 1. Next, the 2´-OH of the last intron residue is thought to initiate the second nucleophilic reaction at the exon 1-intron junction (step 2) resulting in intron circularization and the release of free exon 1. A potential source of free exon 1 is the spliced exon reopening (SER) reaction where both excised lariats and linear introns can recognize and hydrolyze ligated exons at the splice junction. To explain the presence of additional nts at the splice junction of intron circles, the external nucleophilic attack pathway (**d**) was previously proposed [17, 19]. The 3’OH residue of a block of external nts (grey box) attacks the exon 1-intron junction, ligating it to the intron 5’ end while concurrently displacing exon 1 (step 1). The 3’OH at the end of exon 1 then attacks the intron-exon 2 junction releasing ligated exons and a linear intron harboring external nts at its 5’ end (step 2). The third transesterification reaction is initiated by the 2’-OH of the last intron residue (step 3). The position of this final nucleophilic attack thus dictates how many additional nts are incorporated at the junction of intron circles.

Group II introns require the assistance of RNA binding proteins called maturases to adopt their active three-dimensional conformation and self-splice *in vivo* [15]. Specific sequence motifs within group IIA introns mediate the accurate recognition of the 5’ and 3’ splice sites. Exon binding sequence 1 (EBS1) and 2 (EBS2) identify the 5’ splice site by base pairing with complementary intron binding sequence 1 (IBS1) and 2 (IBS2) situated at the 3’ extremity of the upstream exon. The 3’ splice site is recognized by the ∂-∂’ base paring interaction at the 5’ extremity of the downstream exon. Group II introns self-splice from interrupted RNA transcripts through three different splicing pathways (Fig 1) [15]. The branching (Fig 1A), hydrolysis (Fig 1B) and circularization (Fig 1C) pathways release the intron as either branched structures called lariats, in linear forms or as closed circles, respectively. Each of these three splicing pathways involve two consecutive transesterification reactions (Fig 1, steps 1 and 2). Branching, however, is the only splicing pathway that is completely reversible where intron lariats can recognize single- and double-stranded nucleic acid substrates (RNA/DNA) through base pairing and reinsert themselves by reverse splicing (Fig 1A, double arrows) [15, 16]. Since reverse splicing is the initial step of both group II intron mobility pathways, retrohoming and retrotransposition, only released intron lariats are active mobile elements [16].

We recently unveiled and characterized at the molecular level the circularization pathway of Ll.LtrB, the model group II intron, from the gram-positive bacterium *Lactococcus lactis* [17, 18]. Our work showed that the intron excises simultaneously through the branching and circularization pathways *in vivo* leading to the accumulation of both intron lariats and circles respectively. While the majority of the excised intron circles were found to have their 5’ and 3’ ends perfectly joined, we identified Ll.LtrB RNA circles harboring additional nucleotides at their splice junction. Here we describe novel group II intron splicing pathways in which the release of intron circles, harboring or not mRNA fragments of various lengths at their splice junctions, occurs concurrently with the generation of intergenic E1-mRNA and mRNA-mRNA chimeras *in vivo*. Overall, this study unveils that, similarly to spliceosomal introns in eukaryotes, bacterial group II introns generate genetic diversity at the RNA level, producing novel splicing products with potential new functions.

## Results

### Some excised Ll.LtrB RNA circles harbor mRNA fragments of various lengths at their splice junction

**Fig 2:**
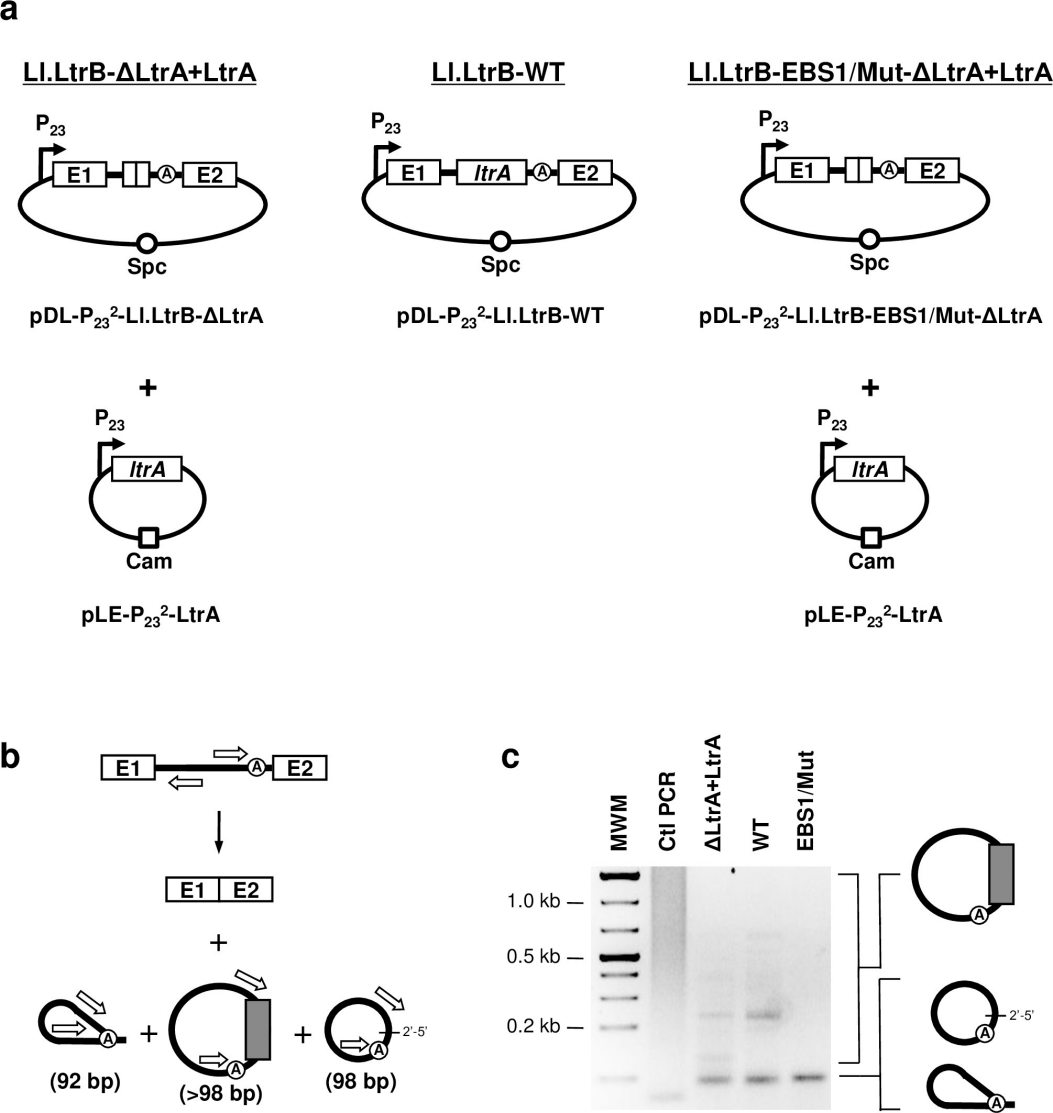
Detection of mRNA fragments at the splice junction of excised intron RNA circles. (**a**) Various Ll.LtrB constructs used in this study where the LtrA protein is provided either in *trans* (Ll.LtrB-ΔLtrA+LtrA, Ll.LtrB-EBS1/Mut-ΔLtrA+LtrA) or in *cis* (Ll.LtrB-WT) (**b**) Schematic of Ll.LtrB self-splicing. Position of the primers (open arrows)(S2 Table) used to amplify the splice junction of excised introns by RT-PCR is depicted (92 bp (lariat), 98 bp (circle) or >98 bp (circle harboring additional nts)). (**c**) RT-PCR amplifications of intron splice junctions. Amplifications were performed on total RNA extracts from *L*. *lactis* (NZ9800Δ*ltrB*) harboring different Ll.LtrB constructs expressed under the control of the P_23_ constitutive promoter (ΔLtrA+LtrA: pDL-P_23_^2^-Ll.LtrB-ΔLtrA and pLE-P_23_^2^-LtrA)(WT: pDL-P_23_^2^-Ll.LtrB-WT)(EBS1/Mut: pDL-P_23_^2^-Ll.LtrB-EBS1/Mut-ΔLtrA and pLE-P_23_^2^-LtrA). The EBS1/Mut intron variant was shown to splice accurately and efficiently *in vivo* by RT-PCR amplifications of both released introns and ligated exons. Additional nts incorporated at the junction of intron circles are represented by a gray box.

**Fig 3:**
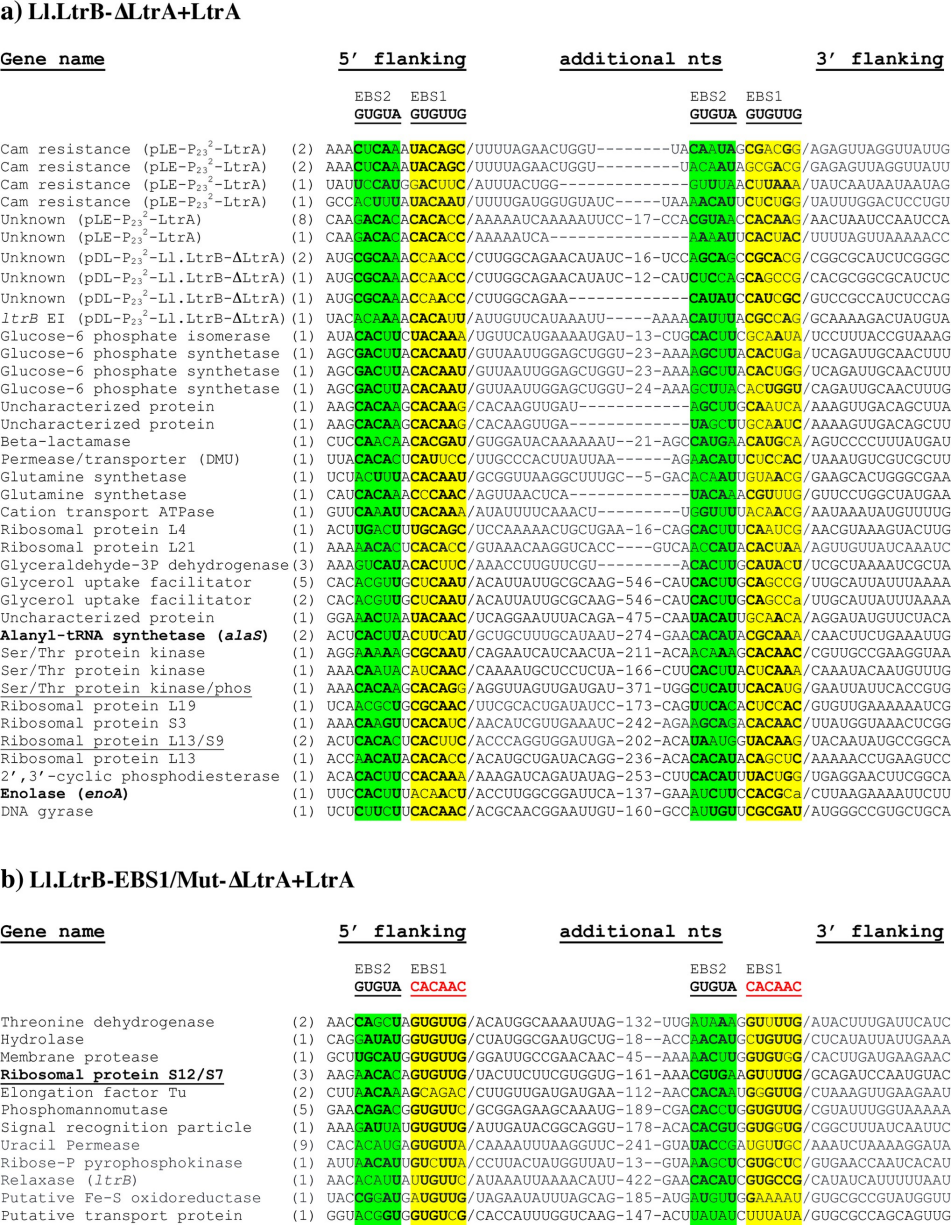
mRNA fragments identified at the splice junction of Ll.LtrB circles. Additional nts are shown along with their flanking sequences (5’ flanking) (3’ flanking), their origin (Gene name) and frequency of identification between parentheses for Ll.LtrB-ΔLtrA+LtrA (a) and Ll.LtrB-EBS1/Mut-ΔLtrA+LtrA (b) circles. The junctions between the additional nts and their flanking regions (/) as well as the IBS1- (yellow) and IBS2- (green) like sequences are denoted. The bolded nts represent residues from the IBS1- and IBS2-like sequences that can potentially base pair with the intron’s EBS1 and EBS2 sequences specified above. Sequences spanning two genes and including a short intergenic region are underlined. The genes in bold (*alaS*, *enoA*, *S12/S7*) were further studied for Ll.LtrB reverse splicing analyses and the detection of E1-mRNA and mRNA-mRNA chimeras (Figs 7 and 8).

To study the splicing pathway leading to the incorporation of additional nucleotides at the splice junction of group II intron circles [17] we performed an RT-PCR reaction across the Ll.LtrB-ΔLtrA+LtrA lariat and circle splice junctions (Fig 2) [17, 18]. We cloned and sequenced the amplicons located in the faint smear above the RT-PCR band that corresponds to perfect lariat and circle splice junctions (Fig 2C). They revealed excised intron circles harboring additional nucleotides (nts) between the first and the last nts of the intron (Fig 3A). The stretch of additional nts greatly varied in size (20–576 nts), originated from the *L*. *lactis* chromosome or the two plasmids used to express the intron (Fig 2A) [17, 18] and mapped to the transcribed strand of annotated genes. Some sequences were identified more than once while others corresponded to different portions of the same gene.

Additional nts within the same size range (26–593 nts) and with identical characteristics (S1 Fig) were identified at the circle splice junction of Ll.LtrB-WT (Fig 2C). Taken together, these data show that mRNA fragments are incorporated at the splice junction of Ll.LtrB RNA circles during circularization, regardless if LtrA, the intron-encoded protein, is expressed in *trans* (Fig 3A) or in *cis* (S1 Fig).

### IBS1/2-like sequences are present upstream of both extremities of the mRNA fragments incorporated at the Ll.LtrB circle splice junction

**Fig 4:**
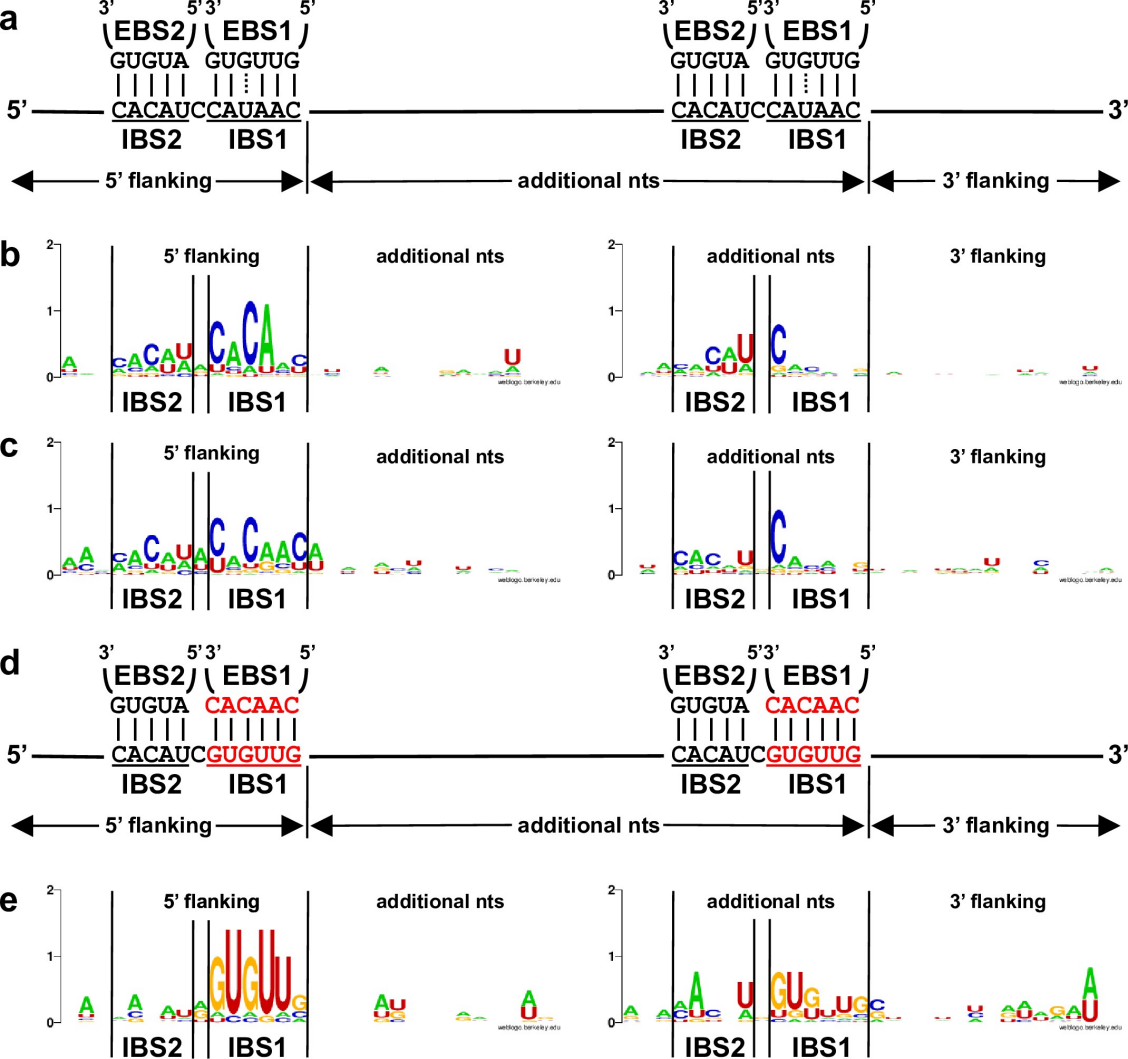
Logo representation of the consensus sequences (30 nts) around the 5’ and 3’ extremities of the mRNA fragments identified at intron circle splice junctions. The EBS1-IBS1 and EBS2-IBS2 base pairing interactions for Ll.LtrB-ΔLtrA+LtrA and Ll.LtrB-WT (**a**) as well as Ll.LtrB-EBS1/Mut-ΔLtrA+LtrA (**d**) are depicted. The consensus sequences are shown for Ll.LtrB-ΔLtrA+LtrA (Fig 3A)(**b**), Ll.LtrB-WT (S1 Fig)(**c**) and Ll.LtrB-EBS1/Mut-ΔLtrA+LtrA (Fig 3B)(**e**).

The flanking sequences on both sides of the mRNA fragments incorporated at the Ll.LtrB-ΔLtrA+LtrA (Fig 3A) and Ll.LtrB-WT (S1 Fig) circle splice junctions were retrieved, compiled and analyzed. Directly upstream from the 5’ and 3’ junctions we identified IBS1/2-like sequences partly complementary to the EBS1/2 sequences for both introns (Figs 3A and S1). Consensus sequences of 30 nts spanning the 5’ and 3’ junctions of the mRNA fragments confirmed the presence of IBS1/2-like sequence motifs. The IBS1-like motifs are better defined than the IBS2-like motifs, whereas the upstream IBS1/2-like motifs are stronger for both Ll.LtrB-ΔLtrA+LtrA and Ll.LtrB-WT (Fig 4A–4C).

### Ll.LtrB recognizes both extremities of the mRNA fragments present at intron circle splice junctions through base pairing

Comparable mRNA fragments of various lengths (43–452 nts)(Fig 3B) were also found at the circle splice junction of Ll.LtrB-EBS1/Mut-ΔLtrA+LtrA (Fig 2C), for which the EBS1 sequence was modified from 5’-GUUGUG-3’ to 5’-CAACAC-3’ (Fig 4D). Accordingly, the IBS1-like consensus sequence motifs upstream from both mRNA junctions were found to be different from Ll.LtrB-ΔLtrA+LtrA and Ll.LtrB-WT and complementary to the mutated EBS1 sequence (Fig 4E). In addition, similarly to Ll.LtrB-ΔLtrA+LtrA and Ll.LtrB-WT, the IBS1-like sequence motifs are both better defined than the IBS2-like motifs and the upstream IBS1/2-like motif much stronger.

The base pairing potential of Ll.LtrB-EBS1/Mut-ΔLtrA+LtrA is more stringent than Ll.LtrB-ΔLtrA+LtrA and Ll.LtrB-WT because its EBS1 sequence (5’-CAACAC-3’) can perfectly recognize only 1 sequence (5’-GUGUUG-3’). In contrast, both introns harboring the wild-type EBS1 sequence (5’-GUUGUG-3’) can base pair perfectly with 64 different sequence combinations using G = U wobble base pairings. Consequently, the more stringent EBS1 sequence led to a fainter RT-PCR smear (Fig 2C), the identification of fewer mRNA fragments at the intron circle splice junction (Fig 3B), and to much stronger flanking consensus motifs when compared to Ll.LtrB-ΔLtrA+LtrA and Ll.LtrB-WT (Fig 4). These data confirm that both junctions of the incorporated mRNA fragments at intron circle splice junctions are recognized by the EBS1/2 motifs of Ll.LtrB through base pairing interactions during circularization.

Consensus sequences are slightly but consistently stronger when flexibility is allowed at both junctions of the mRNA fragments for all three constructs suggesting that Ll.LtrB does not always process mRNAs precisely downstream from the recognized IBS1/2-like motifs (S2–S5 **Figs**). We also identified mRNA fragments, at intron circle splice junctions, that either contained untranslated sequences or spanned two genes including the short intergenic regions of polycistronic mRNAs (**Figs** 3 and S1). This further supports our conclusion that Ll.LtrB can capture *L*. *lactis* transcripts at intron circle splice junctions during circularization.

### Models of mRNA fragment incorporation at group II intron circle splice junctions

**Fig 5:**
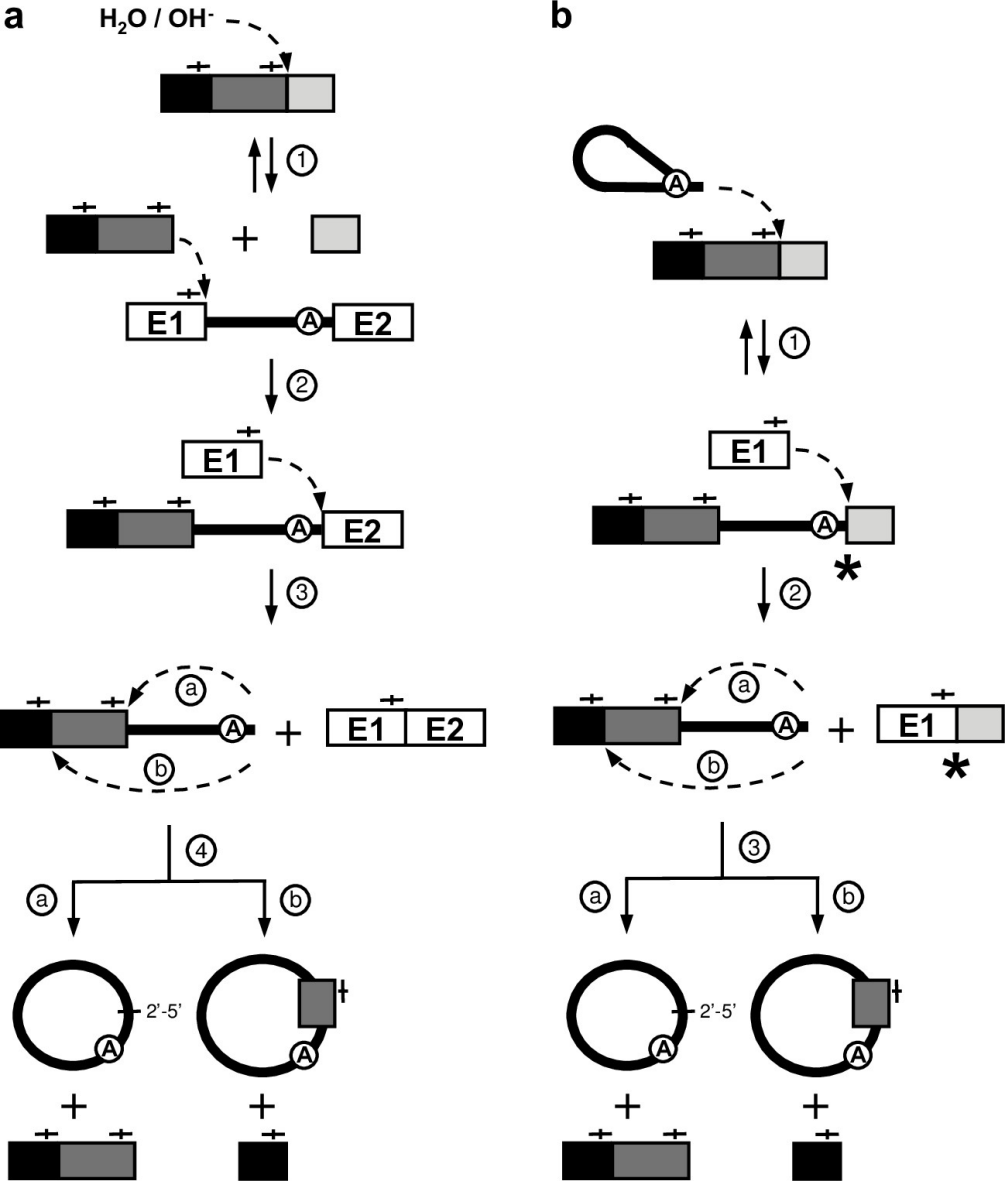
Models for the incorporation of mRNA fragments at the splice junction of intron RNA circles. (**a**) External nucleophilic attack pathway [17, 19]. The Ll.LtrB group II intron recognizes, through base pairing interactions, an IBS1/2-like sequence (—|—) on an mRNA and guides the first nucleophilic attack induced by an hydroxyl ion or a water molecule downstream of the recognized sequence (step 1). Next, the 3’-OH of the processed mRNA induces a nucleophilic attack at the exon 1-intron splice junction resulting in its ligation to the 5’ end of the intron and the release of exon 1 (step 2). The 3’-OH of exon 1 is then free to initiate the second transesterification reaction at the intron-exon 2 splice junction, releasing ligated exons and a linear intron harboring a fragment of mRNA at its 5’ end (step 3). The final transesterifictaion reaction is induced at the intron 5’ end (a) or within the mRNA (b) by the 2’-OH of the last nt of the linear intron, just downstream from IBS1/2-like sequences (—|—), resulting in the release of either a head-to-tail circular intron (step 4a) or an intron circle harboring an mRNA fragment at its splice junction (step 4b). (**b**) Reverse splicing pathway. This pathway is initiated by the reverse splicing of an intron lariat within a non-cognate mRNA downstream of an IBS1/2-like sequence (—|—)(step 1). The 3’-OH of free exon 1 then attacks the phosphodiester bond at the 3’ splice site between the last nt of the intron and the 3’ segment of the mRNA (step 2). This generates a chimeric mRNA consisting of the *ltrB*-exon 1 (E1) linked to the 3’ segment of the mRNA (E1-mRNA) and a circularization intermediate where the linear intron is still attached to the 5’ segment of the mRNA. The third transesterifictaion reaction is induced at the intron 5’ end (a) or within the mRNA fragment (b) by the 2’-OH of the last residue of the linear intron, just downstream from IBS1/2-like sequences (—|—), resulting in the release of either a head-to-tail circular intron (step 3a) or an intron circle harboring an mRNA fragment at its splice junction (step 3b). The 3’ junction of reverse-spliced introns and the chimeric E1-mRNAs are unique splicing intermediates that distinguish both pathways (asterisks).

Our findings indicate that cellular mRNAs can somehow be incorporated at the Ll.LtrB circle splice junction during the circularization pathway. Two models can explain how mRNA fragments could be incorporated at the splice junction of group II intron circles (Fig 5).

The external nucleophilic attack pathway (Fig 5A) was previously proposed to explain how short stretches of additional nts could be incorporated at the circle splice junction during intron circularization. However, the pathway of integration and the origin of the additional nts were never demonstrated [17, 19]. Taking into consideration the data presented here, Ll.LtrB would recognize, through base pairing interactions, an IBS1/2-like sequence on an *L*. *lactis* mRNA and guide its hydrolysis downstream of the recognized sequence (step 1). Next, the 3’-OH of the processed mRNA would induce a transesterification reaction at the exon 1-intron splice junction resulting in its ligation to the 5’ end of the intron and the release of exon 1 (step 2). The 3’-OH of exon 1 would then initiate the next transesterification reaction at the intron-exon 2 splice junction, releasing ligated exons and a linear intron harboring an mRNA fragment at its 5’ end (step 3). The final transesterifictaion reaction would be induced at the intron 5’ end (step 4a) or within the mRNA (step 4b) by the 2’-OH of the last nt of the linear intron, just downstream from IBS1/2-like sequences, resulting in the release of either a head-to-tail circular intron or an intron circle harboring an mRNA fragment at its splice junction respectively.

An alternative pathway (Fig 5B) would rather be initiated by the reverse splicing of an intron lariat within an *L*. *lactis* mRNA downstream of an IBS1/2-like sequence (step 1). The ectopically inserted group II intron would then excise from the mRNA through circularization (steps 2–4). The 3’-OH of free exon 1 would first attack the phosphodiester bond at the 3’ splice site between the last nt of the intron and the 3’ segment of the mRNA (step 2). This would generate a chimeric mRNA consisting of the *ltrB*-exon 1 (E1) linked to the 3’ segment of the mRNA (E1-mRNA) and a circularization intermediate where the linear intron is still attached to the 5’ segment of the mRNA. The final transesterifictaion reaction would then be induced at the intron 5’ end (step 3a) or within the mRNA fragment (step 3b) by the 2’-OH of the last nt of the intron, just downstream from IBS1/2-like sequences, resulting in the release of either a head-to-tail circular intron or an intron circle harboring an mRNA fragment at its splice junction respectively.

### Ll.LtrB lariats reverse splice within *L*. *lactis* mRNAs downstream of IBS1/2-like sequences

**Fig 6:**
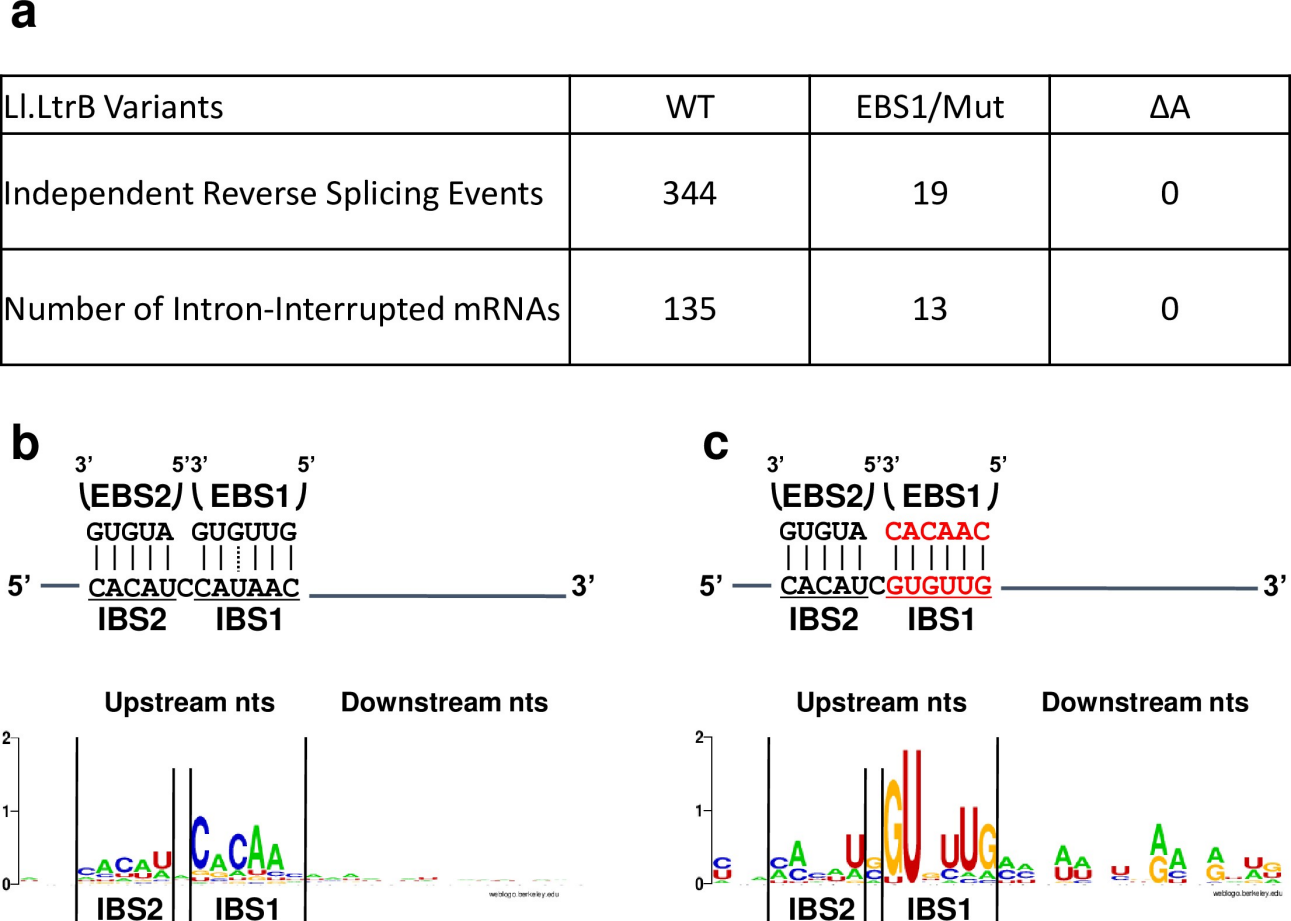
Ll.LtrB reverse splicing within *L*. *lactis* mRNAs. (**a**) Independent Ll.LtrB reverse splicing events were identified by total RNA-Seq for Ll.LtrB-ΔLtrA+LtrA, Ll.LtrB-EBS1/Mut-ΔLtrA+LtrA and the control Ll.LtrB-ΔA-ΔLtrA+LtrA that exclusively splices through the circularization pathway and cannot reverse splice. The EBS1-IBS1 and EBS2-IBS2 base pairing interactions for Ll.LtrB-ΔLtrA+LtrA (**b**) and Ll.LtrB-EBS1/Mut-ΔLtrA+LtrA (**c**) are depicted. Logo representations of the consensus sequences upstream (15 nts) and downstream (15 nts) from the intron reverse splice sites within the various *L*. *lactis* mRNAs are also shown.

To investigate the proposed models we looked for unique intermediates of the reverse splicing pathway: the 3’ junction of Ll.LtrB reverse-spliced within mRNAs and chimeric E1-mRNAs (Fig 5B, asterisks). We first detected by RNA-Seq intron-interrupted mRNAs for Ll.LtrB-ΔLtrA+LtrA and Ll.LtrB-EBS1/Mut-ΔLtrA+LtrA but not for the Ll.LtrB-ΔA-ΔLtrA+LtrA control which lacks the essential branch point A residue required for branching and reverse splicing [17, 18, 20] (Fig 6A). The reverse splice sites of Ll.LtrB-ΔLtrA+LtrA (Fig 6B) and Ll.LtrB-EBS1/Mut-ΔLtrA+LtrA (Fig 6C) were shown to be immediately preceded by consensus IBS1/2-like sequence motifs complementary to their respective EBS1/2 sequences. On the other hand, similarly to the junctions between intron circles and mRNA fragments (**Figs** 4 and S5), we did not detect a ∂’-like sequence on the 3’ side of the intron insertion sites (Fig 6). This shows that Ll.LtrB can recognize IBS1/2-like sequences on various mRNAs by base pairing with its EBS1/2 sequences and invade them by reverse splicing, generating a population of intron-interrupted mRNAs in *L*. *lactis*. As expected, the more stringent EBS1 sequence of Ll.LtrB-EBS1/Mut-ΔLtrA+LtrA led to the identification of fewer intron-interrupted mRNAs and a stronger IBS1/2-like consensus sequence upstream of the intron insertion sites compared to Ll.LtrB-ΔLtrA+LtrA.

**Fig 7:**
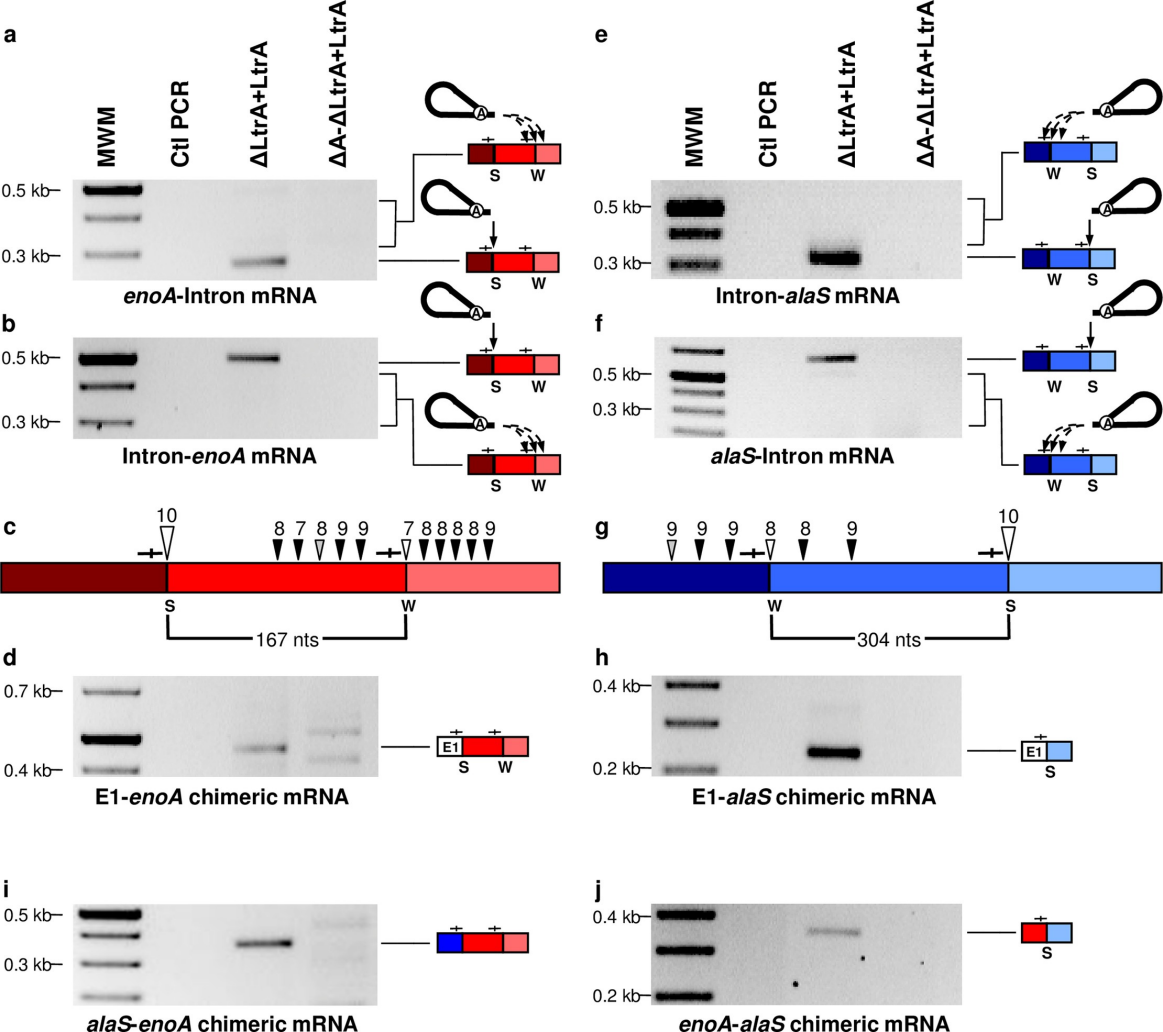
Detection of intermediates unique to the reverse splicing pathway: Ll.LtrB reverse-spliced within *L*. *lactis* mRNAs, E1-mRNA and mRNA-mRNA chimeras. RT-PCR assays were performed to detect the 5’ and 3’ junctions of Ll.LtrB-ΔLtrA+LtrA and Ll.LtrB-ΔA-ΔLtrA+LtrA reverse splicing events within the *enoA* (red boxes)(**a**, **b**) and *alaS* (blue boxes)(**e**, **f**) mRNAs. Complete and dashed arrows indicate reverse splicing of Ll.LtrB lariats within strong (S) and weak (W) IBS1/2-like sequences (—|—) respectively. The strong (S)(10/11 nts)(large arrowhead) and weak (W)(7-9/11 nts)(small arrowhead) IBS1/2-like sequences invaded by reverse splicing are represented (**c**, **g**). The sites flanking the mRNA fragments (**c**, 167 nts and **g**, 304 nts) initially detected at intron circle splice junctions (Fig 3A) are indicated by open arrowheads. The Ll.LtrB insertion sites were identified in conditions where the *enoA* or the *alaS* genes were overexpressed (small open and black arrowheads) or not (large open and small gray arrowheads) from a P_23_ constitutive promoter. mRNA chimeras between *ltrB*-exon 1 (E1) and *L*. *lactis* mRNAs (**d**, E1-*enoA*)(**h**, E1-*alaS*) as well as between *L*. *lactis* mRNAs (**i**, *alaS-enoA*)(**j**, *enoA-alaS*) were also detected by RT-PCR at IBS1/2-like sequences.

**Fig 8:**
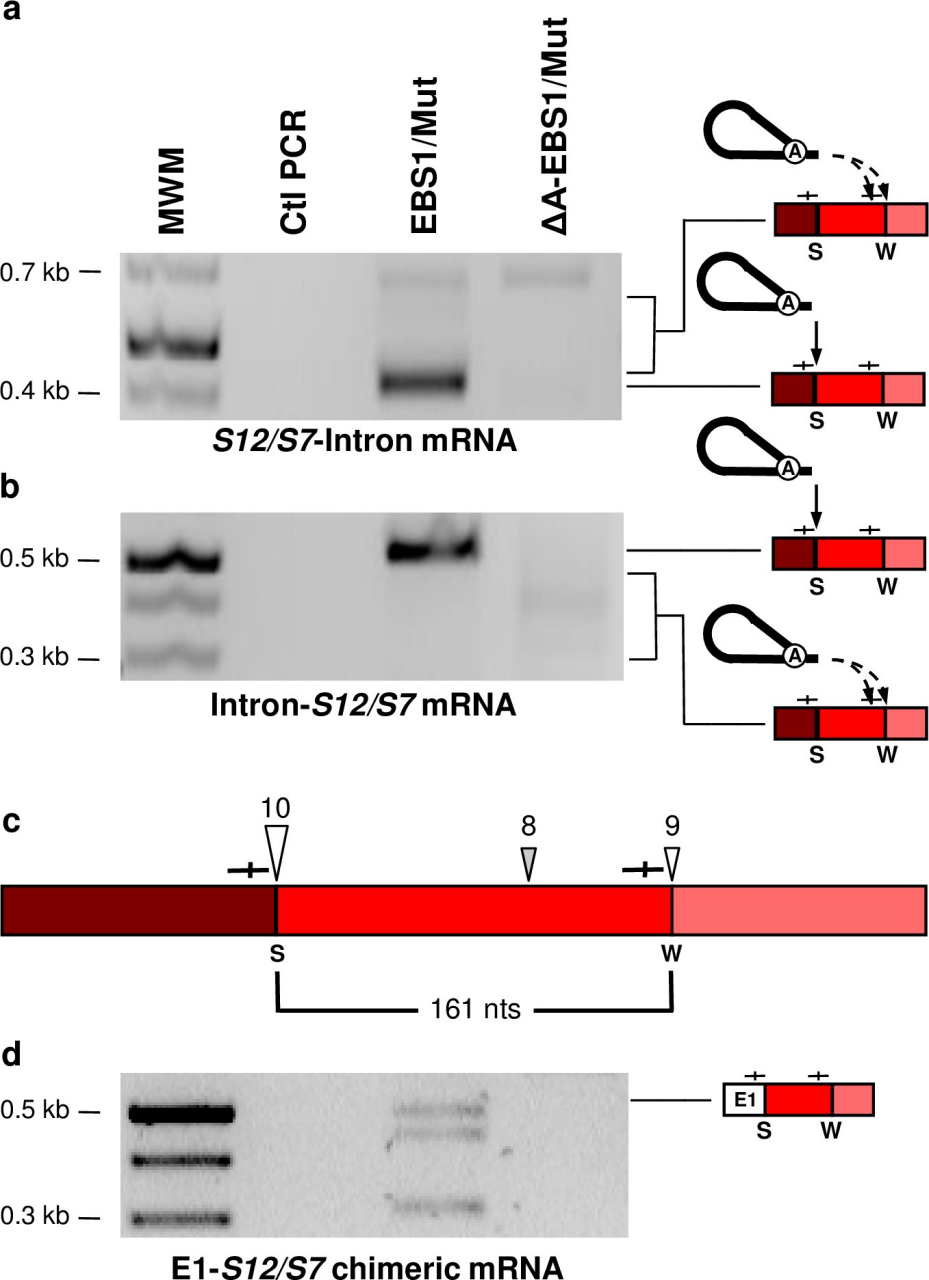
Detection of intermediates unique to the reverse splicing pathway: Ll.LtrB reverse-spliced within an *L*. *lactis* mRNA and an E1-mRNA chimera. RT-PCR assays were performed to detect the 5’ and 3’ junctions of Ll.LtrB-EBS1/Mut-ΔLtrA+LtrA and Ll.LtrB-ΔA-EBS1/Mut-ΔLtrA+LtrA reverse splicing events within the Ribosomal Protein *S12/S7* mRNA (**a**, **b**). Complete and dashed arrows indicate reverse splicing of Ll.LtrB lariats within strong (S) and weak (W) IBS1/2-like sequences (—|—) respectively. The strong (S)(10/11 nts)(large arrowhead) and weak (W)(8-9/11 nts)(small arrowheads) IBS1/2-like sequences invaded by reverse splicing are represented (**c**). The sites flanking the mRNA fragment (**c**, 161 nts) initially detected at intron circle splice junctions (Fig 3B) are indicated by open arrowheads. mRNA chimeras between *ltrB*-exon 1 (E1) and *L*. *lactis* mRNAs (**d**, E1-*S12/S7*) were also detected by RT-PCR at IBS1/2-like sequences.

We next studied in further details the reverse splicing of Ll.LtrB-ΔLtrA+LtrA within the Enolase (*enoA*) and Alanyl-tRNA synthetase (*alaS*) mRNAs. The *enoA* (167 nts) and *alaS* (304 nts) mRNA fragments, previously identified at the Ll.LtrB-ΔLtrA+LtrA circle splice junction, are both flanked by a strong (10/11 nts) and a weak (7/11 and 8/11 nts) IBS1/2-like sequence motif (Fig 3A). We amplified by RT-PCR the 5’ (Fig 7A and 7F) and 3’ (Fig 7B and 7E) junctions between the intron and the two mRNAs. Sequences of the four amplicons confirmed reverse splicing of the intron precisely downstream of the strong IBS1/2-like sequence within the *enoA* (Fig 7C, large open arrowhead) and *alaS* (Fig 7G, large open arrowhead) mRNAs. Importantly, no amplifications were detected for the reverse splicing deficient control, Ll.LtrB-ΔA-ΔLtrA+LtrA. Next, the faint smears above (Fig 7A and 7E) and below (Fig 7B and 7F) the main amplicons were cloned and shown to correspond to several independent 5’ and 3’ junctions of the intron inserted downstream of different weak IBS1/2-like sequences (7-9/11 nts)(Fig 7C and 7G, black and grey arrowheads). The weak IBS1/2-like sequences flanking the mRNA fragments previously identified within intron circles (Fig 3A), were also found invaded by the intron for both *enoA* (Fig 7C, small open arrowhead) and *alaS* (Fig 7G, small open arrowhead). Similarly, the Ll.LtrB-EBS1/Mut-ΔLtrA+LtrA variant was shown to reverse splice at specific strong and weak IBS1/2-like sequences within the *S12/S7* transcript (Fig 8A–8C). The identified reverse splice sites also include the strong and weak IBS1/2-like sequences flanking the *S12/S7* mRNA fragment (161 nts) previously identified at the intron circle splice junction (Fig 3B).

Collectively, these results show that IBS1/2-like sequences are widespread within *L*. *lactis* mRNAs, providing abundant targets for Ll.LtrB reverse splicing. They also support the proposed alternative circularization model by which introns, reverse-spliced at ectopic sites within mRNAs, can circularize alternatively by recognizing upstream IBS1/2-like sequences leading to the capture of mRNA fragments at their splice junction (Fig 5B, step 3b). Accordingly, when additional nts are found at intron circle splice junctions, the upstream IBS1/2-like consensus sequences are consistently stronger (Figs 4 and S5) suggesting that when the intron reverse splices at a weak IBS1/2-like sequence, it is more likely to release intron circles harboring mRNA fragments by recognizing a stronger upstream alternative IBS1/2-like sequence.

### Ll.LtrB circularization from intron-interrupted mRNAs generates E1-mRNA and mRNA-mRNA chimeras

The second distinguishing splicing intermediate between the two proposed models is a chimeric mRNA consisting of *ltrB*-exon 1 (E1) *trans*-spliced to an *L*. *lactis* mRNA fragment (E1-mRNA) (Fig 5B, asterisk). We specifically screened for E1-*enoA* and E1-*alaS* mRNA chimeras by RT-PCR. In both cases we detected, exclusively for the reverse splicing-competent intron, E1-mRNA chimeras ligated precisely downstream from the strong IBS1/2-like sequences (Fig 7D and 7H), the exact sites previously identified at one of the extremities of the mRNA fragments identified at intron circle splice junctions (Fig 3A) and invaded by reverse splicing (Fig 7A, 7B, 7E and 7F). The intron-catalyzed EBS1/2-specific generation of E1-mRNA chimeras was corroborated with the Ll.LtrB-EBS1/Mut-ΔLtrA+LtrA variant again at the previously identified strong IBS1/2-like sequence of the *S12/S7* transcript (Fig 8D). These results show that Ll.LtrB, reverse-spliced at IBS1/2-like sequences of various mRNAs, can recruit free E1 through EBS-IBS base pairing interactions, and catalyze the formation of E1-mRNA chimeras.

**Fig 9:**
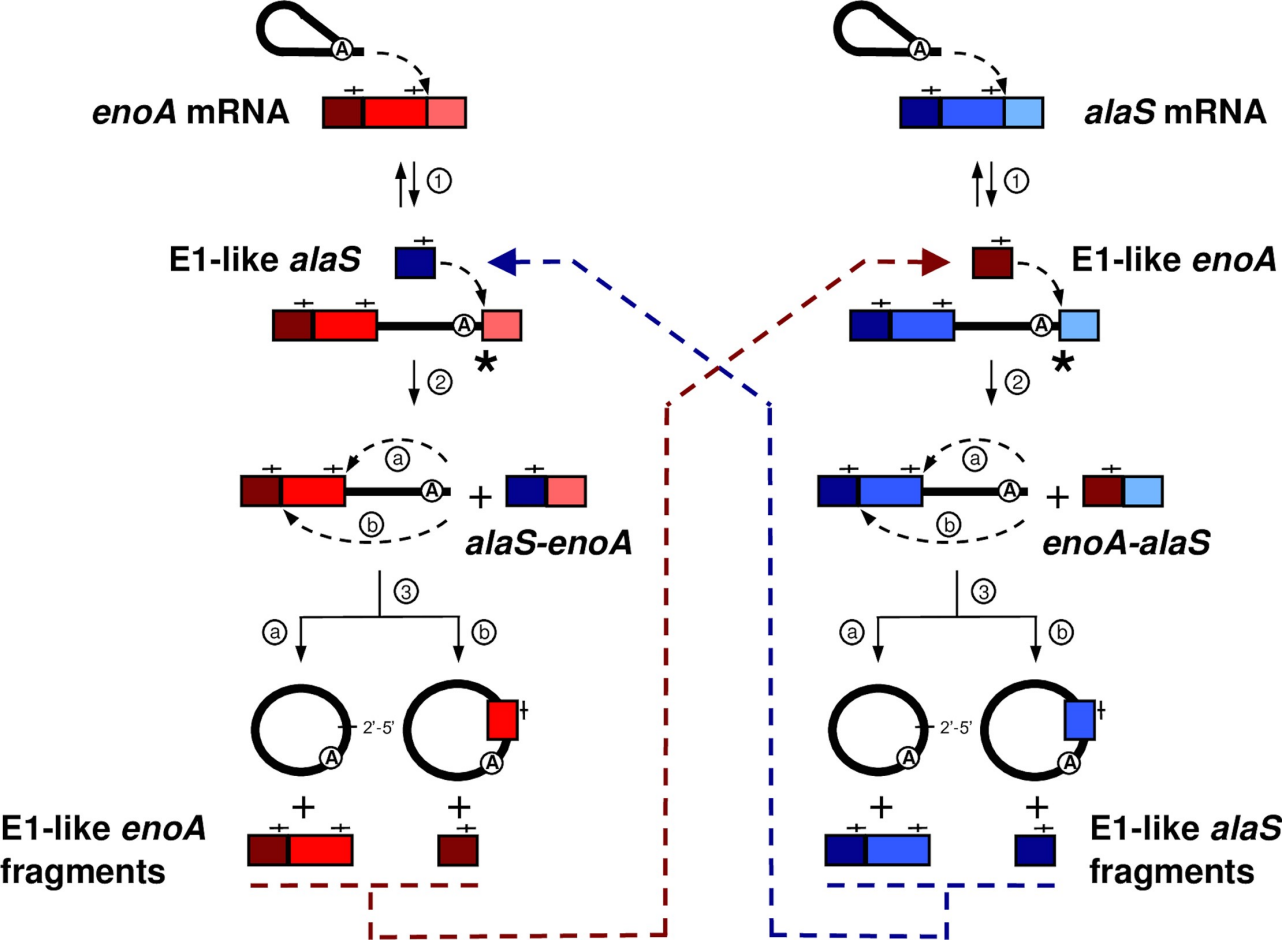
The intergenic group II intron *trans*-splicing pathway leading to the generation of *alaS-enoA* and *enoA-alaS* mRNA chimeras. Ll.LtrB first recognizes by base pairing and invades by reverse splicing IBS1/2-like sequences on the *enoA* (red) and *alaS* (blue) mRNAs (step 1). The 3’OH of intron-processed *alaS* mRNA fragments (E1-like *alaS* fragments), harboring IBS1/2-like sequences (—|—) at their 3’ ends (dark blue and blue)(dark blue), can, similarly to free E1, be recruited by the intron to initiate the circularization pathway. These fragments can attack the intron-exon 2 splice junction of the Ll.LtrB-interrupted *enoA* mRNA (red) leading to the generation of *alaS-enoA* mRNA chimeras (step 2). Subsequent excision of the intron by circularization releases *enoA* mRNA fragments (E1-like *enoA* fragments) with IBS1/2-like sequences at their 3’ ends (step 3a, dark red and red)(step 3b, dark red), which can in turn initiate the generation of *enoA-alaS* mRNA chimeras with intron-interrupted *alaS* mRNA (blue).

Ll.LtrB splicing *via* circularization, from a population of intron-interrupted mRNAs, generates processed mRNA fragments harboring IBS1/2-like sequences at their 3’ end (Fig 5B, step 3a and 3b). We next examined if these splicing products could be recruited by Ll.LtrB, similarly to free E1 through EBS-IBS base pairing, and used to generate intergenic mRNA-mRNA chimeras (Fig 9). We detected by RT-PCR both *alaS*-*enoA* (Fig 7I) and *enoA*-*alaS* (Fig 7J) mRNA-mRNA intergenic chimeras joined at specific IBS1/2-like sequences for Ll.LtrB-ΔLtrA+LtrA but not for the Ll.LtrB-ΔA-ΔLtrA+LtrA control. These data show that Ll.LtrB, reverse-spliced within various mRNAs, can recruit through base pairing processed mRNA fragments, harboring IBS1/2-like sequences at their 3’ end, to initiate the circularization splicing pathway (Fig 9, step 2). Ll.LtrB can thus catalyze the shuffling of coding sequences within a population of intron-interrupted mRNAs by a new intergenic *trans*-splicing pathway (Fig 9).

## Discussion

One quarter of currently sequenced bacterial genomes harbor one to a few group II introns [10]. This paucity, coupled with their irregular distribution and frequent lateral transfer [4], has led to the suggestion that they are selfish retromobile elements with no beneficial function to their host [5]. In contrast, many group II intron derivatives provide important functions in both eukaryotes and prokaryotes [1–3]. For example, the abundant spliceosomal introns, descendants of group II introns, generate significant genetic diversity and transcriptomic complexity *via* alternative splicing [21], intergenic *trans*-splicing [22], RNA circle formation [23] and by creating new genes through exon shuffling [24].

Even though the Ll.LtrB group II intron is present at only one copy in the *L*. *lactis* genome, the new splicing pathways described here (**Figs** 5B and 9) expand the genetic diversity and complexity of its host transcriptome. This stems from the ability of Ll.LtrB, following its release as RNP particles, to generate a population of intron-interrupted mRNAs through reverse splicing, which we were able to detect by RNA-Seq (Fig 6) and gene-specific RT-PCR (**Figs** 7 and 8). Ll.LtrB was recently shown to interact with its cognate ligated exons at the IBS1/2 site *in vivo*, leading to either complete reverse splicing or negative regulation of targeted mRNA through hydrolysis and degradation [25]. However, when we contrasted the counts per million (CPM) of Ll.LtrB-WT, Ll.LtrB-EBS1/Mut-ΔLtrA+LtrA and Ll.LtrB-ΔA-ΔLtrA+LtrA constructs for *alaS*, the most abundant target for reverse splicing that we identified by RNA-Seq, we obtained differential expression ratios that showed very little change in the abundance of the *alaS* transcript: 0.97 between Ll.LtrB-WT and EBS1/Mut-ΔLtrA+LtrA and 0.96 between Ll.LtrB-WT and Ll.LtrB-ΔA-ΔLtrA+LtrA. This suggests that the IBS1/2-like sites we identified within host mRNAs are not efficient targets for hydrolysis, but rather seem to be used for reverse splicing. Interestingly, several of the reverse-splicing sites found by RNA-Seq were also identified independently at the extremity of mRNA fragments captured at intron circle splice junctions (**Figs** 3A and S1), yet there was only a small overlap of IBS1/2-like motifs between these two sets of data. Moreover, when we analysed the *enoA* and *alaS* genes in greater detail, we found a multitude of additional IBS1/2-like motifs that were used as targets for Ll.LtrB reverse splicing and whose base paring interactions with the intron varied from strong (11/11 nts) to weak (7/11 nts) (Fig 7C and 7G). Overall, our data thus suggest that the reverse-splicing of group II introns into ectopic sites within host mRNAs is a widespread, dynamic and transient process whose exact scope is hard to determine.

**Fig 10:**
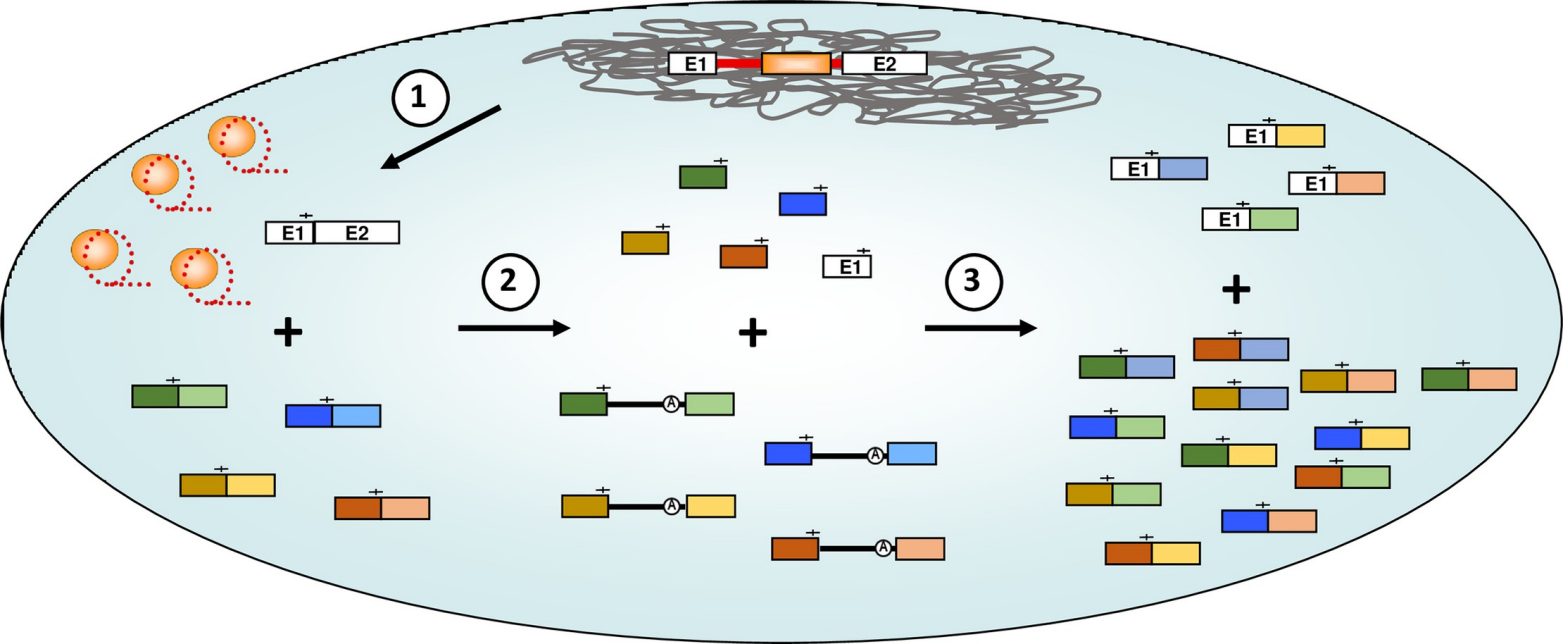
Model for group II intron-catalysed genetic diversity. Upon expression of group II intron-interrupted genes in bacteria, the ribozymes self-splice using the conventional branching pathway, releasing a mix of RNPs (lariats + LtrA) and accurately ligated flanking exons (step 1). Excised RNPs next interact with cellular mRNA transcripts through specific base pairing with IBS1/2-like sequences (—|—). This interaction leads either to complete reverse splicing or hydrolysis at the IBS1/2-like sites, producing a population of intron-invaded mRNA transcripts or hydrolysed mRNA fragments with a free 3’-OH, respectively (step 2). When introns interrupting an ectopic site self-splice using the circularization pathway, they recruit either processed ectopic mRNA fragments or their processed cognate E1, which can both act as external nucleophiles in an intergenic *trans*-splicing reaction (step 3). This produces two distinct populations of chimeric mRNA transcripts: E1-mRNA and mRNA-mRNA products, which together increase the overall diversity of the bacterial host’s transcriptome. The presence of a series of group II intron–interrupted mRNAs may potentially lead to a multitude of chimeric mRNA-mRNA combinations.

We demonstrated that circularization of Ll.LtrB from interrupted mRNAs, using free E1 or mRNA fragments harboring IBS1/2-like sequences at their 3’ end, generates two types of *trans*-spliced transcripts: E1-mRNA (Fig 5B) and mRNA-mRNA (Fig 9) chimeras respectively. Ll.LtrB was recently found to generate free E1 *in vivo* through hydrolysis of ligated cognate exons at the IBS1/2 site [25]. This Spliced Exon Reopening (SER) reaction (Fig 1) could thus produce the initial source of E1 required to initiate Ll.LtrB circularization from both its cognate exons and ectopic insertion sites. In addition, we found that alternative circularization of Ll.LtrB from interrupted mRNAs releases intron circles harboring mRNA fragments at their splice junction (Fig 5B, step 3b). These novel bacterial splicing products, generated by alternative circularization and intergenic *trans*-splicing, may have and/or lead to novel biological functions for their host cell. For instance, chimeric RNAs, intron circles and different circular RNAs that accumulate *in vivo* have been recently associated to a variety of interesting new functions such as RNA sponges, protein sponges and transcriptional regulators in various biological systems [23, 26, 27]. Moreover, the *trans*-spliced E1-mRNA and mRNA-mRNA chimeras could be reclaimed by the host and potentially lead to the creation of new genes. Group II introns may thus serve a beneficial function for their hosts by increasing the complexity and genetic diversity of their transcriptomes (Fig 10) which could explain why they were retained in bacteria.

Our work also unveils two additional functional and evolutionary links between group II introns, spliceosomal introns and the spliceosome. First, the *trans*-splicing of E1 at the 5’ end of various mRNA fragments is analogous to the second step of the spliced leader (SL) *trans*-splicing pathway, which has a patchy evolutionary distribution amongst eukaryotes and whose origin has remained enigmatic [28, 29]. Second, we showed that group II introns, similarly to the spliceosome [22], can catalyze the *trans*-splicing of intergenic mRNA-mRNA chimeras in bacteria. Since group II introns are considered as the progenitors of both spliceosomal introns and the snRNAs of the spliceosome [1–3], our findings suggest that the spliceosome-dependent formation of SL *trans*-spliced transcripts and intergenic mRNA-mRNA chimeras in eukaryotes both consist of ancient group II intron splicing functions still shared with their contemporary bacterial relatives.

Overall, we described here new group II intron splicing pathways that generate and expand the genetic diversity and complexity of its host transcriptome which represents a new function for these bacterial retroelements. Our work also unveils new functional and evolutionary links with their nuclear relatives in eukaryotes, and provide a potential explanation of why group II introns were maintained in bacteria.

## Materials and methods

### Bacterial strains and plasmids

*Lactococcus lactis* strain NZ9800Δ*ltrB* (Tet^R^) [8] was grown in M17 media supplemented with 0.5% glucose (GM17) at 30°C without shaking. The *Escherichia coli* strain DH10β, used for cloning purposes, was grown in LB at 37°C with shaking. Antibiotics were used at the following concentrations: chloramphenicol (Cam^R^), 10 μg/ml; spectinomycin (Spc^R^), 300 μg/ml. Previously constructed plasmids (pDL-P_23_^2^-Ll.LtrB-ΔLtrA [30], pDL-P_23_^2^-Ll.LtrB-WT [30], pLE-P_23_^2^-LtrA [31]) were used to study Ll.LtrB splicing. Additional variants were constructed by site-directed mutagenesis (New England Biolabs Q5 Site-Directed-Mutagenesis Kit): pDL-P_23_^2^-Ll.LtrB-ΔA-ΔLtrA, pDL-P_23_^2^-Ll.LtrB-EBS1/Mut-ΔLtrA. The alanyl tRNA synthetase (*alaS*) and enolase (*enoA*) genes were cloned in pLE-P_23_^2^-LtrA (BssHII), downstream of the second P_23_ promoter, and expressed with the intron in a two-plasmid system. Primers used for mutagenesis and cloning are in S2 Table.

### RNA extraction, PCR and RT-PCR

Total RNA was isolated from NZ9800Δ*ltrB* harboring various plasmid constructs as previously described [31]. RT-PCR reactions [17, 18] were performed on total RNA preparations of NZ9800Δ*ltrB* harboring various intron constructs (primers in S2 Table).

### RNA-Seq

RNA-Seq was performed on rRNA-depleted total RNA from *L*. *lactis* (NZ9800Δ*ltrB*) expressing Ll.LtrB-ΔLtrA+LtrA, Ll.LtrB-ΔA-ΔLtrA+LtrA, or Ll.LtrB-EBS1/Mut-ΔLtrA+LtrA using the Illumina HiSeq 2500 paired-end sequencing system [32].

### Sequence consensus

Aligned and adjusted consensuses were prepared using the WebLogo software [33]. Adjusted consensuses were determined using a code that calculated contiguous nucleotides with the highest capacity of base pairing to EBS1 and EBS2 (total of 11 nucleotides), separated from each other by 0–2 nucleotides, in a region that spanned -14, +4 nts around the junction with the intron.

## Supporting information

S1 Fig mRNA fragments identified at the splice junction of Ll.LtrB-WT circles. (PDF)

S2 Fig mRNA fragments identified at the splice junction of Ll.LtrB-ΔLtrA+LtrA circles. (PDF)

S3 Fig mRNA fragments identified at the splice junction of Ll.LtrB-WT circles. (PDF)

S4 Fig mRNA fragments identified at the splice junction of Ll.LtrB-EBS1/Mut-ΔLtrA+LtrA circles. (PDF)

S5 Fig Logo representation of the consensus sequences (30 nts) around the 5’ and 3’ extremities of the mRNA fragments identified at intron circle splice junctions. (PDF)

S1 Table Plasmids. (PDF)

S2 Table Primers. (PDF)
