# Supplementary material for: Bacterial group II introns generate genetic diversity by circularization and trans-splicing from a population of intron-invaded mRNAs
Source: PLoS Genet. 2018 Nov 21;14(11):e1007792. doi: 10.1371/journal.pgen.1007792 (PMC6248898; doi:10.1371/journal.pgen.1007792)
Supplement: S1 Fig — (PDF) [file pgen.1007792.s001.pdf]

Ll.LtrB-WT

Gene name		5′ flanking	additional nts	3′ flanking
		EBS2 EBS1<br>**GUGUA** **GUGUUG**	EBS2 EBS1<br>**GUGUA** **GUGUUG**	
5′nucleotidase	(2)	ACACACUUCCACAAA	/AAAGAUCAGAUAUAG-253-CUUCACAUUUACUGG	/UGAGGAACUUCGGCA
a-acetolactate decarboxylase	(1)	CAAUACAUUUAGCAC	/ACUCUCUAGCGGCUU-26--AAGCACUAACACACG	/GCAAAGUCGGUAUCG
Ribosomal protein L13/S9	(1)	ACCAACAUACACACC	/ACAUGCUGAUACAGG-431-GCAGACAUGCGUCUU	/GUUAUCAACCAACCA
Ribosomal protein L13/S9	(1)	ACACACACAUACAGC	/UCAAAAACCUGAAGU-355-UACACGUGACGCCCG	/UAUGGUUGAACGUAA
Ribosomal protein L13/S9	(2)	ACCAACAUACACACC	/ACAUGCUGAUACAGG-303-ACAUAAUGGCACAAG	/UACAAUAUGCCGGCA
L-lactate dehydrogenase	(1)	UGCAGCUUACUCAAU	/CAUCGCUAAAAAAGG-170-UUCACAUUCCAUUGA	/ACGAUGCUGAAAUGC
NADH dehydrogenase	(1)	AACAGCACACACAGC	/AGCGGCAAAUAUUCU-563-CUCAUAAAGCAACUG	/AAUGGGGUCUCUCCA
ABC transporter permease	(1)	AUGCACUUUCCCAAU	/UUUCAUCUGUCCGUA-150-UUUCACCUGCUUCCA	/ACAGCCUUAGUACAU
Glutamine synthetase	(1)	CAUCACAAACCCAAC	/AGUUAACUCAUACAA-386-CAAGCUAUGCACAAU	/UUGUAUCGCAAUGGG
Glyceraldehyde dehydrogenase	(1)	AAUGUCAUACACUUC	/AAACCUUGUUC---------GUACACUUGCAUACU	/UCGCUAAAAUCGCUA
Elongation factor Tu	(1)	ACUCCAGAACGCGAC	/ACUGACAAACCACUC-362-CUACUUCCACACAAC	/UGACGUUACUGGUUC
Ribosomal protein L19	(1)	ACUGAUAUCCCUGAC	/UUCCGUCCUGGUGAC-153-GUUCACACUCCACGU	/GUUGAAAAAAUCGAA
Ribosomal protein L11	(4)	AUUCACAUUCAUCAC	/AAAAACUCCUCCAGC-233-CCUCACCUACUUAGG	/UAGGCUUUGGAACCU
N-acetylglucosaminidase	(1)	GACAACAAACACAAG	/UAAUACCGCUUCAAG-173-GCACAUCAGCAUCAU	/CUGGUAGUUACACAA
Aldehyde dehydrogenase	(1)	CCACACGUCCACAAA	/GCUUUCAUUGUUGCC-254-UUCCGAUGACGCAAG	/UUUGAAAGAGAUCUU
Phosphotransferase	(1)	UAUCUGGUACACAAC	/UUCAUGAGAUUCUUG-94--UAUCACAUUCCUUUG	/UAUGUGGUUGCUUAA
Ferredoxin oxidoreductase	(1)	GACCCCUUAUACAAC	/AAAUGAACAGGAACA-107-AAGCACUUCCAGAAA	/UGUCGGAUGAUUUAC
ABC transporter	(1)	UGAACAUAUUGCGAU	/UCUAGGCCGUUCAGG-136-UAUCUCUUUGCAACA	/ACGGUUCGUUCUAAU
g-aminobutyrate permease	(1)	ACUCAUUAUCGCAAC	/UUAAUGAUGAUAAAA-56--UUCACAUCACUAUUG	/AUUUUAUUUACUCCA
Uncharacterized prot 1-2	(1)	UAGACCUUCCACGAU	/UUAUAGUAUACCUAG-214-AACCACAGAAACAAU	/UAAAAUAUUGCUACA
Thioredoxin reductase	(1)	AAUCAUUAUCGCGAC	/UGGAGCUAAUCACCG-185-AAUUACGUGCACAAG	/AAAUUAUUCAACAAA
Arsenate reductase	(1)	CACAAUCUAUACAGC	/ACCCUCUUGUACAAG-185-UUUCACUUUCACAAG	/CAAUUAAAAUCAUUU
NADH dehydrogenase	(1)	AACAGCACACACAGC	/AGCGGCAAAUAUUCU-173-CAGCACAUUAUUUCU	/GGCGUUAUGCCUUGG
Transcriptional regulator	(1)	UAAAACGCACACGAC	/AACAAUGGUUUCAAU-173-ACAGCUUUGCAACUG	/UAAAUUUUGAUGGUU
Uncharacterized protein	(1)	AAACACAAGUCUACU	/GGAAAAUAUAUCAAA-284-UGACUCAGGCACCAA	/AAAAGAAAGCAGCAA
Ser/Thr protein kinase	(1)	AACAAUACAUCAACC	/AAAAUGCUCCUCUAG-51--UGGCUCAUUCACAUG	/GAAUUAUUCACCGUG
a-acetolactate decarboxylase	(1)	CAAUACAUUUACGAC	/ACUCUCUAGCGGCUU-50--GUAUCGGUACGCUUG	/AUACGGCAAAUGGCG
Ribosomal protein S14	(1)	AAUACAUAAAUGGCU	/AAGAAAUCUAUGGUU-64--GUCCACAUUCAGUUU	/ACCGCAAAUUUAAAC
N-acetylglucosaminidase	(1)	UAACUCAAACUCGAC	/UUCUUCUAACUCAAA-50--AAAAAUCUGGCAGCC	/CAAUUGCUUCAAUCA

Figure S1. mRNA fragments identified at the splice junction of Ll.LtrB-WT circles. Additional nts are shown along with their flanking sequences (5' flanking) (3' flanking), their origin (Gene name) and frequency of identification between parentheses. The junctions between the additional nts and their flanking regions (/) as well as the IBS1- (yellow) and IBS2- (green) like sequences are denoted. The bolded nts represent residues from the IBS1- and IBS2-like sequences that can potentially base pair with the intron's EBS1 and EBS2 sequences specified above. Sequences spanning two genes and including a short intergenic region are underlined while the mRNA sequence including 3' untranslated residues is italicized.
