# Supplementary material for: Bacterial group II introns generate genetic diversity by circularization and trans-splicing from a population of intron-invaded mRNAs
Source: PLoS Genet. 2018 Nov 21;14(11):e1007792. doi: 10.1371/journal.pgen.1007792 (PMC6248898; doi:10.1371/journal.pgen.1007792)
Supplement: S2 Fig — (PDF) [file pgen.1007792.s002.pdf]

Ll.LtrB-ΔLtrA+LtrA

Gene name		5′ flanking / additional nts / 3′ flanking
		EBS2 **GUGUA** EBS1 **GUGUUG** ... EBS2 **GUGUA** EBS1 **GUGUUG**
Cam resistance (pLE-P_{23}^{2}-LtrA)	(2)	AAACUCAAAUACAGC/UUUUAGAACUGGU--------UACAAUAGCGACGG/AGAGUUAGGUUAUUG
Cam resistance (pLE-P_{23}^{2}-LtrA)	(2)	AAACUCAAAUACAGC/UUUUAGAACUGGU---------UACAAUAGCGACG/GAGAGUUAGGUUAUU
Cam resistance (pLE-P_{23}^{2}-LtrA)	(1)	UAUUCCAUGGACUUC/AUUUACUGG--------------GUUUAACUUAAA/UAUCAAUAAUAAUAG
Cam resistance (pLE-P_{23}^{2}-LtrA)	(1)	GCCACUUUAUACAAU/UUUUGAUGGUGUAUC-----UAAAACAUUCUCUGG/UAUUUGGACUCCUGU
Unknown (pLE-P_{23}^{2}-LtrA)	(8)	CAAGACACACACACC/AAAAAUCAAAAAUUCC-17-CCACGUAACCACAAG/AACUAAUCCAAUCCA
Unknown (pLE-P_{23}^{2}-LtrA)	(1)	CAAGACACACACACC/AAAAAUCA---------------AAAAUUCACUAC/UUUUAGUUAAAAACC
Unknown (pDL-P_{23}^{2}-Ll.LtrB-ΔLtrA)	(2)	AUGCGCAAACCAACC/CUUGGCAGAACAUAUC-16-UCCAGCAGCCGCACG/CGGCGCAUCUCGGGC
Unknown (pDL-P_{23}^{2}-Ll.LtrB-ΔLtrA)	(1)	AUGCGCAAACCAACC/CUUGGCAGAACAUAUC-12-CAUCUCCAGCAGCCG/CACGCGGCGCAUCUC
Unknown (pDL-P_{23}^{2}-Ll.LtrB-ΔLtrA)	(1)	AUGCGCAAACCAACC/CUUGGCAGAA-------------CAUAUCCAUCGC/GUCCGCCAUCUCCAG
ltrB EI (pDL-P_{23}^{2}-Ll.LtrB-ΔLtrA)	(1)	UACACAAAACACAUU/AUUGUUCAUAAAUU-----AAAACAUUUACGCCAG/GCAAAAGACUAUGUA
Glucose-6 phosphate isomerase	(1)	AUACACUUCUACAAA/UGUUCAUGAAAAUGAU-13-CUGCACUUCGCAAUA/UCCUUUACCGUAAAG
Glucose-6 phosphate synthetase	(1)	AGCGACUUACACAAU/GUUAAUUGGAGCUGGU-23-AAAAGCUUACACUGa/UCAGAUUGCAACUUU
Glucose-6 phosphate synthetase	(1)	AGCGACUUACACAAU/GUUAAUUGGAGCUGGU-23-AAAAGCUUACACUGG/UCAGAUUGCAACUUU
Glucose-6 phosphate synthetase	(1)	AGCGACUUACACAAU/GUUAAUUGGAGCUGGU-24-AAAGCUUACACUGGU/CAGAUUGCAACUUUG
Uncharacterized protein	(1)	AAGCACAAGCACAAG/CACAAGUUGA------------UAGCUUGCAAUCA/AAAGUUGACAGCUUA
Uncharacterized protein	(1)	AAGCACAAGCACAAG/CACAAGUUGA-------------UAGCUUGCAAUC/AAAAGUUGACAGCUU
Beta-lactamase	(1)	CUCCAACAACACGAU/GUGGAUACAAAAAAU--21-AGCCAUGAACAUGCA/AGUCCCCUUUAUGAU
Permease/transporter (DMU)	(1)	UUACACACUCAUUCC/UUGCCCACUUAUUAA------AGAACAUUCUCCAC/UAAAUGUCGUCGCUU
Glutamine synthetase	(1)	UCUACUUUACACAAU/GCGGUUAAGGCUUUGC--5-GACACAAUUGUAACG/GAAGCACUGGGCGAA
Glutamine synthetase	(1)	CAUCACAAACCCAAC/AGUUAACUCA-------------UACAAACGUUUG/GUUCCUGGCUAUGAA
Cation transport ATPase	(1)	GUUCAAAUUCACAAA/AUAUUUUCAAACU---------UGGUUUUACAACG/AAUAAAUAUGUUUUG
Ribosomal protein L4	(1)	ACUUGACUUUGCAGC/UCCAAAAACUGCUGAA-16-CAGCACUUUCAAUCG/AACGUAAAGUACUUG
Ribosomal protein L21	(1)	AAAAACACUCACACC/GUAAACAAGGUCACC----GUCAACCAUACACUAA/AGUUGUUAUCAAAUC
Glyceraldehyde-3P dehydrogenase	(3)	AAAGUCAUACACUUC/AAACCUUGUUCGU---------ACACUUGCAUACU/UCGCUAAAAUCGCUA
Glycerol uptake facilitator	(5)	CACACGUUGCUCAAU/ACAUUAUUGCGCAAG-546-CAUCACUUGCAGCCG/UUGCAUUAUUUAAAA
Glycerol uptake facilitator	(2)	CACACGUUGCUCAAU/ACAUUAUUGCGCAAG-546-CAUCACUUGCAGCCa/UUGCAUUAUUUAAAA
Uncharacterized protein	(1)	GGAAACUAAUACAAC/UCAGGAAUUUACAGA-475-CAAUACAUUGCAACA/AGGAUAUGUUCUACA
**Alanyl-tRNA synthetase (*alaS*)**	(2)	ACUCACUUACUUCAU/GCUGCUUUGCAUAAU-274-GAACACAUACGCAAA/CAACUUCUGAAAUUG
Ser/Thr protein kinase	(1)	AGGAAAAAGCGCAAU/CAGAAUCAUCAACUA-211-ACAACAAAGCACAAC/CGUUGCCGAAGGUAA
Ser/Thr protein kinase	(1)	AAACAAUACAUCAAC/CAAAAUGCUCCUCUA-166-CUUCACUUACUCAAA/CAAAUACAAUGUUUG
Ser/Thr protein kinase/phos	(1)	AAACACAAGCACAGG/AGGUUAGUUGAUGAU-371-UGGCUCAUUCACAUG/GAAUUAUUCACCGUG
Ribosomal protein L19	(1)	UCAACGCUGCGCAAC/UUCGCACUGAUAUCC-173-CAGUUCACACUCCAC/GUGUUGAAAAAAUCG
Ribosomal protein S3	(1)	AAACAAGUUCACAUC/AACAUCGUUGAAAUC-242-AGAAGCAGACACAAC/UUAUGGUAAACUCGG
Ribosomal protein L13/S9	(2)	ACUCACACUCACUUC/ACCCAGGUGGAUUGA-202-ACAUAAUGGUACAAG/UACAAUAUGCCGGCA
Ribosomal protein L13	(1)	ACCAACAUACACACC/ACAUGCUGAUACAGG-236-ACACACAUACAGCUC/AAAAACCUGAAGUCC
2′,3′-cyclic phosphodiesterase	(1)	ACACACUUCCACAAA/AAAGAUCAGAUAUAG-253-CUUCACAUUUACUGG/UGAGGAACUUCGGCA
**Enolase (*enoA*)**	(1)	UUCCACUUUACAACU/ACCUUGGCGGAUUCA-137-GAAAUCUUCCACGCa/CUUAAGAAAAUUCUU
DNA gyrase	(1)	UCUCUUCUUCACAAC/ACGCAACGGAAUUGU-160-GCCAUUGUUCGCGAU/AUGGGCCGUGCUGCA

Figure S2. mRNA fragments identified at the splice junction of Ll.LtrB-ΔLtrA+LtrA circles. Additional nts are shown along with their flanking sequences (5’ flanking) (3’ flanking), their origin (Gene name) and frequency of identification between parentheses. The junctions between the additional nts and their flanking regions (/) as well as the IBS1- (yellow) and IBS2- (green) like sequences are denoted. Some IBS1/2-like sequences were adjusted to optimize their potential base pairing with the EBS1/2 sequences of the intron. The number of nts separating the IBS1/2-like sequences was fixed between 0-2 nts, and their maximum distance from the junction with the intron was fixed between -14, +4 nts. The bolded nts represent residues from the IBS1- and IBS2-like sequences that can potentially base pair with the intron’s EBS1 and EBS2 sequences specified above. Sequences spanning two genes and including a short intergenic region are underlined. The genes in bold (*alaS* and *enoA*) were further studied for Ll.LtrB reverse splicing analyses and the detection of E1-mRNA and mRNA-mRNA chimeras (Figure 7).
