# Supplementary material for: Bacterial group II introns generate genetic diversity by circularization and trans-splicing from a population of intron-invaded mRNAs
Source: PLoS Genet. 2018 Nov 21;14(11):e1007792. doi: 10.1371/journal.pgen.1007792 (PMC6248898; doi:10.1371/journal.pgen.1007792)
Supplement: S4 Fig — (PDF) [file pgen.1007792.s004.pdf]

Ll.LtrB-EBS1/Mut-ΔLtrA+LtrA

```
Gene name                        5' flanking          additional nts          3' flanking

                                   EBS2  EBS1                                  EBS2  EBS1
                                   GUGUA CACAAC                                GUGUA CACAAC

Threonine dehydrogenase       (2) AACCAGCUAGUGUUG/ACAUGGCAAAAUUAG-132-UUGAUAAAGGUUUUG/AUACUUUGAUUCAUC
Hydrolase                     (1) CAGGAUAUGGUGUUG/CUAUGGCGAAUGCUG-18--ACCAACAUGCUGUUG/CUCAUAUUAUUGAAA
Membrane protease             (1) GCUUGCAUGGUGUUG/GGAUUGCCGAACAAC-45--AAAAACUUGGUGUGG/CACUUGAUGAAGAAC
Ribosomal protein S12/S7      (3) AAGAACACAGUGUUG/UACUUCUUCGUGGUG-161-AAACGUGAAGUUUUG/GCAGAUCCAAUGUAC
Elongation factor Tu          (2) CUUAACAAAGCAGAC/CUUGUUGAUGAUGAA-112-AACCACAAUGGGUUG/CUAAAGUUGAAGAAU
Phosphomannomutase            (5) GAACAGACGGUGUUC/GCGGAGAAGCAAAUG-189-CGACACCUGGUGUUG/CGUAUUUGGUAAAAA
Signal recognition particle   (1) AAAGAUUAUGUGUUG/AUUGAUACGGCAGGU-178-ACACACGUGGUGGUG/CGGCUUUAUCAAUUC
Uracil Permease               (9) CACACAUGAGUGUUA/CAAAAUUUAAGGUUC-241-GUAUACCGAUGUUGC/AAAUCUAAAAGGAUA
Ribose-P pyrophosphokinase    (1) AUUAACAUUGUCUUA/CCUUACUAUGGUUAU-13--GUAAAGCUCGUGCUC/GUGAACCAAUCACAU
Relaxase (ltrB)               (1) AACACAUUAUUGUUC/AUAAAUUAAAACAUU-422-GAACACAUCGUGCCG/CAUAUCAUUUUUAAU
Putative Fe-S oxidoreductase  (1) UACCGGAUGAUGUUG/UAGAAUAUUUAGCAG-185-AUGAUGUUGGAAAAU/GUGCGCCGUAUGGUU
Putative transport protein    (1) GGUACGGUGGUGUCG/CACCAUUUGGUCAAG-147-ACUUAUAUCUUUAUA/GUGCGCCAGCAGUUG
```

Figure S4. mRNA fragments identified at the splice junction of Ll.LtrB-EBS1/Mut-ΔLtrA+LtrA circles. Additional nts are shown along with their flanking sequences (5' flanking) (3' flanking), their origin (Gene name) and frequency of identification between parentheses. The junctions between the additional nts and their flanking regions (/) as well as the IBS1- (yellow) and IBS2- (green) like sequences are denoted. Some IBS1/2-like sequences were adjusted to optimize their potential base pairing with the EBS1/2 sequences of the intron. The number of nts separating the IBS1/2-like sequences was fixed between 0-2 nts, and their maximum distance from the junction with the intron was fixed between -14, +4 nts. The bolded nts represent residues from the IBS1- and IBS2-like sequences that can potentially base pair with the intron's EBS1 and EBS2 sequences specified above. Sequence spanning two genes and including a short intergenic region is underlined. The gene in bold (*S12/S7*) was further studied for Ll.LtrB reverse splicing analyses and the detection of E1-mRNA chimeras (Figure 8).
