# Supplementary material for: Bacterial group II introns generate genetic diversity by circularization and trans-splicing from a population of intron-invaded mRNAs
Source: PLoS Genet. 2018 Nov 21;14(11):e1007792. doi: 10.1371/journal.pgen.1007792 (PMC6248898; doi:10.1371/journal.pgen.1007792)
Supplement: S5 Fig — (PDF) [file pgen.1007792.s005.pdf]

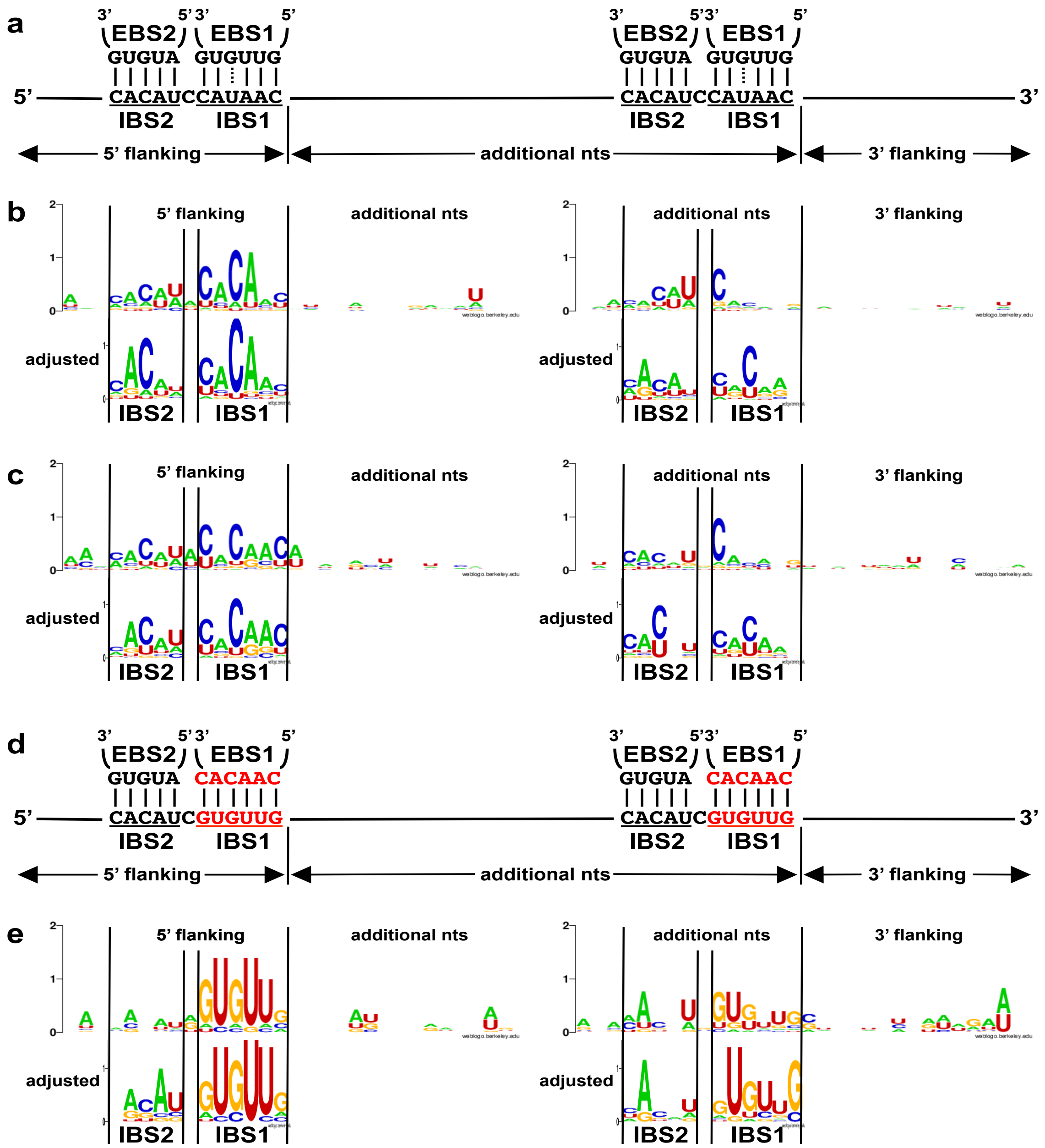

Figure S5. Logo representation of the consensus sequences (30 nts) around the 5' and 3' extremities of the mRNA fragments identified at intron circle splice junctions. The EBS1-IBS1 and EBS2-IBS2 base pairing interactions for Ll.LtrB-ΔLtrA+LtrA and Ll.LtrB-WT (**a**) as well as Ll.LtrB-EBS1/Mut-ΔLtrA+LtrA (**d**) are depicted. The consensus (Figure 3, S1) and adjusted consensus (Figure S2-S4) sequences are shown for Ll.LtrB-ΔLtrA+LtrA (**b**), Ll.LtrB-WT (**c**) and Ll.LtrB-EBS1/Mut-ΔLtrA+LtrA (**e**).
