# Supplementary material for: Bacterial group II introns generate genetic diversity by circularization and trans-splicing from a population of intron-invaded mRNAs
Source: PLoS Genet. 2018 Nov 21;14(11):e1007792. doi: 10.1371/journal.pgen.1007792 (PMC6248898; doi:10.1371/journal.pgen.1007792)
Supplement: S1 Table — (PDF) [file pgen.1007792.s006.pdf]

Table S1. Plasmids

Plasmid name	Size and antibiotic resistance	Description
pDL-$P_{23}{}^2$-Ll.LtrB-WT	11 kb, Spc^R (300µg/µl)	*E. coli-L. lactis* shuttle vector harboring wild-type Ll.LtrB expressed under the control of the P_{23} constitutive promoter
pDL-$P_{23}{}^2$-Ll.LtrB-ΔLtrA	9.2 kb, Spc^R (300µg/µl)	Ll.LtrB lacking its essential intron-encoded protein (LtrA)
pDL-$P_{23}{}^2$-Ll.LtrB-ΔA-ΔLtrA	9.2 kb, Spc^R (300µg/µl)	Ll.LtrB lacking LtrA and the branch point adenosine residue
pDL-$P_{23}{}^2$- Ll.LtrB-EBS1/Mut-ΔLtrA	9.2 kb, Spc^R (300µg/µl)	Ll.LtrB lacking LtrA and mutated in the EBS1 (intron) and IBS1 (exon1) regions to modify base pairing specificity
pDL-$P_{23}{}^2$- Ll.LtrB-ΔA-EBS1/Mut-ΔLtrA	9.2 kb, Spc^R (300µg/µl)	Ll.LtrB lacking LtrA, the branch point adenosine and mutated in the EBS1 (intron) and IBS1 (exon1) regions to modify base pairing specificity
pLE-$P_{23}{}^2$-LtrA	10.9 kb, Cam^R (10µg/µl)	*E. coli-L. lactis* shuttle vector harboring the ltrA gene expressed under the control of the P_{23} constitutive promoter
pLE-$P_{23}{}^2$-LtrA+AlaS	11.7 kb, Cam^R (10µg/µl)	Alanyl-tRNA synthetase gene (*alaS*) from *L. lactis* expressed under the control of the P_{23} constitutive promoter
pLE-$P_{23}{}^2$-LtrA+EnoA	10.4 kb, Cam^R (10µg/µl)	Enolase gene (*enoA*) from *L. lactis* expressed under the control of the P_{23} constitutive promoter
