# Supplementary material for: Bacterial group II introns generate genetic diversity by circularization and trans-splicing from a population of intron-invaded mRNAs
Source: PLoS Genet. 2018 Nov 21;14(11):e1007792. doi: 10.1371/journal.pgen.1007792 (PMC6248898; doi:10.1371/journal.pgen.1007792)
Supplement: S2 Table — (PDF) [file pgen.1007792.s007.pdf]

**Table S2.** Primers

| Primer location | Function | Sequence (5'-3') |
|---|---|---|
| Ll.LtrB 5' end (-) strand | RT of splice junction and PCR of 5' junctions of reverse splicing | CGATTGTCTTTAGGTAACTCAT |
| Ll.LtrB 5' end (+) strand | PCR of circle splice junction | TTAAACTACTTGACTTAACACCC |
| Ll.LtrB 3' end (-) strand | PCR of circle splice junction | TGTGAACAAGGCGGTACCTC |
| *alaS* 5' end (+) strand | Cloning *alaS* gene for overexpression | AAAAGCGCGCGTGGTACCGCGGTATAACTGT |
| *alaS* 3' end (-) strand | Cloning *alaS* gene for overexpression | AAAAGCGCGCCTATCCTAATTTTTCAGCAACAGC |
| *enoA* 5' end (+) strand | Cloning *enoA* gene for overexpression | AAAAGCGCGCTGTATTAAGAGTGCAGACGCAC |
| *enoA* 3' end (-) strand | Cloning *enoA* gene for overexpression | AAAAGCGCGCTTAATGCATTTTTTTAAGGTTGTAGAATGCTTTAA |
| *alaS* 3' end (-) strand | RT to screen for *ltrB* E1-*alaS* chimeras | CCAATCCAGCCACTTCGCTC |
| *alaS* 3' end (-) strand | PCR to screen for *ltrB* E1-*alaS* chimeras | CTGTCACAGCAATAATCCGGC |
| *ltrB*-E1 3' end (+) strand | PCR to screen for *ltrB* E1-*alaS*/*enoA* chimeras | TTGGTCATCACCTCATCCAATC |
| *enoA* 3' end (-) strand | RT to screen for *ltrB* E1-*enoA* chimeras, PCR for 3' junction of Ll.LtrB reverse splicing in *enoA* | CATAACGCCATCTTCACCAGC |
| *enoA* 3' end (-) strand | PCR to screen for *ltrB* E1-*enoA* chimeras | TAACCAGCTGCTTCGATTGCTT |
| *alaS* 3' end (-) strand | RT of 5' and 3' junctions of Ll.LtrB reverse splicing in *alaS* | CAACGATTTGTGAAGCTTGTGG |
| *alaS* 5' end (+) strand | PCR of 5' junction of Ll.LtrB reverse splicing in *alaS* | GGTCAAGTGGTGGCAACTGT |
| *alaS* 3' end (-) strand | PCR of 3' junction of Ll.LtrB reverse splicing in *alaS* | AACCAATCCAGCCACTTCGCT |
| Ll.LtrB 3' end (+) strand | PCR of 3' junction of Ll.LtrB reverse splicing in *alaS* and *enoA* | CTCTTGTTGTATGCTTTCATTG |
| *enoA* 3' end (-) strand | RT of 5' and 3' junctions of Ll.LtrB reverse splicing in *enoA* | GAATTCTGATGATGCACAGTCG |
| *enoA* 5' end (+) strand | PCR of 5' junction of Ll.LtrB reverse splicing in *enoA* | ATGATCGCTCTTGACGGTACT |
| Ll.LtrB 3' end (-) strand | PCR to remove branch point adenosine residue from Ll.LtrB-ΔLtrA | GGCGGTACCTCCCTCTTCACCATATCAT |
| Intron 5' end (+) strand | PCR to mutate the EBS1 sequence of Ll.LtrB | GTAAGTTATGCAACACGACTTATCTGTTATCACCACC |
| Intron 5' end (-) strand | PCR to mutate the EBS1 sequence of Ll.LtrB | CTTTCTTTGTACTAGAGGTTTC |
| *ltrB*-E1 3' end (+) strand | PCR to mutate the IBS1 sequence of *ltrB*-E1 | TGAACACATCGTGTTGGTGCGCCCAGATAGGGTGTTAAG |
| *ltrB*-E1 3' end (-) strand | PCR to mutate the IBS1 sequence of *ltrB*-E1 | CGATCGACGTGGGTTGCA |
